# A literature review and novel theoretical approach on the optical properties of whole blood

**DOI:** 10.1007/s10103-013-1446-7

**Published:** 2013-10-12

**Authors:** Nienke Bosschaart, Gerda J. Edelman, Maurice C. G. Aalders, Ton G. van Leeuwen, Dirk J. Faber

**Affiliations:** Biomedical Engineering and Physics, Academic Medical Center, University of Amsterdam, P.O. Box 22700, 1100 DE Amsterdam, The Netherlands

**Keywords:** Blood, Optical properties, Spectroscopy, Absorption coefficient, Scattering coefficient, Scattering anisotropy

## Abstract

Optical property measurements on blood are influenced by a large variety of factors of both physical and methodological origin. The aim of this review is to list these factors of influence and to provide the reader with optical property spectra (250–2,500 nm) for whole blood that can be used in the practice of biomedical optics (tabulated in the appendix). Hereto, we perform a critical examination and selection of the available optical property spectra of blood in literature, from which we compile average spectra for the absorption coefficient (*μ*_a_), scattering coefficient (*μ*_s_) and scattering anisotropy (*g*). From this, we calculate the reduced scattering coefficient (*μ*_s_′) and the effective attenuation coefficient (*μ*_eff_). In the compilation of *μ*_a_ and *μ*_s_, we incorporate the influences of absorption flattening and dependent scattering (i.e. spatial correlations between positions of red blood cells), respectively. For the influence of dependent scattering on *μ*_s_, we present a novel, theoretically derived formula that can be used for practical rescaling of *μ*_s_ to other haematocrits. Since the measurement of the scattering properties of blood has been proven to be challenging, we apply an alternative, theoretical approach to calculate spectra for *μ*_s_ and *g*. Hereto, we combine Kramers–Kronig analysis with analytical scattering theory, extended with Percus–Yevick structure factors that take into account the effect of dependent scattering in whole blood. We argue that our calculated spectra may provide a better estimation for *μ*_s_ and *g* (and hence *μ*_s_′ and *μ*_eff_) than the compiled spectra from literature for wavelengths between 300 and 600 nm.

## Introduction

The interaction of light with blood plays an important role in optical diagnostics and therapeutics—for instance for the non-invasive assessment of blood composition [1] and the laser treatment of varicose veins [2]. Predictions on the accuracy and outcome of these optical methods can be obtained through simulation models of the light–blood interaction. The reliability of these models depends foremost on accurate knowledge of the optical properties of blood, which include the absorption coefficient *μ*_a_, scattering coefficient *μ*_s_ and scattering anisotropy *g* that parameterizes the phase function *p*(*θ*). Dating back to as early as 1943 [3], many studies have focused on the quantitative assessment of these optical properties [4–10]. These studies demonstrated that optical property measurements on whole blood are challenging, due to the considerable light attenuation in undiluted blood. Although light attenuation is less in diluted samples, rescaling of the optical properties from these samples to whole blood introduces an additional challenge because the scattering properties of blood scale non-linearly as a function of red blood cell concentration (haematocrit) [10–12]. As a consequence, sample preparation, but also measurement method and conditions (e.g. blood flow [7, 13–16]), influences the outcome of the optical property assessment considerably. In this review article, we will therefore provide an overview, interpretation and compilation of the available literature on the optical properties of blood in the visible and near-infrared wavelength range (250–2,500 nm). Our inclusion criteria are (1) publication of both quantitative and spectrally resolved data on *μ*_a_, *μ*_s_ and *g* and (2) the use of human blood from healthy adults for sample preparation.

In part I (‘Methods’ and ‘Results’ sections) of this article, we focus on the absorption coefficient of whole blood. We compile an average *μ*_a_ spectrum for blood with a haematocrit of 45 % from rescaled spectra that are available in literature, while excluding outlier spectra. We also incorporate the effect of ‘absorption flattening’: the phenomenon that the absorption spectrum of a system of strongly absorbing particles (i.e. red blood cells in whole blood) is reduced compared to that of a suspension containing the same number of absorbing molecules in homogeneous dispersion (i.e. haemolysed blood).

The scattering properties of blood (*μ*_s_ and *g*) are considered in part II (‘Theoretical estimation of *μ*_s_ and *g*’, ‘Methods’ and ‘Results’ sections) of this article. Given the difficulty in measuring the scattering properties of red blood cells, and the relative ease of measuring absorption spectra of the red blood cells’ contents, we previously proposed a computational approach based on a Kramers–Kronig analysis of the complex refractive index of haemoglobin [17]. We obtained estimates of red blood cell scattering by combining this approach with analytical scattering theory. Here, we extend this method using Percus–Yevick structure factors that take into account the spatial correlations between the positions of individual red blood cells in a whole blood medium. From this, we obtain calculated spectra of *μ*_s_ and *g* for oxygenized and deoxygenized blood. Moreover, we present a novel scaling relation for *μ*_s_ to different haematocrit values, which we use to theoretically verify a previously published empirical scaling relation [11]. We apply the novel scaling relation to rescale the available literature spectra for *μ*_s_ to a haematocrit of 45 %. From the rescaled spectra, we compile an average *μ*_s_ spectrum for whole blood. We also provide a compiled spectrum of the literature spectra of *g*. To provide the reader with reasonable means to estimate the scattering coefficient, we present an empirical power law for scattering coefficient versus wavelength (>700 nm). In addition, we provide spectra for the reduced scattering coefficient (*μ*_s_′) and the effective attenuation coefficient (*μ*_eff_), derived from both the compiled and calculated spectra of *μ*_a_, *μ*_s_ and *g*.

The main results of this article are ready-to-use compiled spectra of *μ*_a_, as well as both compiled and calculated spectra of *μ*_s_, *μ*_s_′, *μ*_eff_ and *g* for whole blood with a haematocrit of 45 %. For convenience, these spectra are tabulated in the Appendix of this article. Moreover, methods for scaling between different haematocrits are presented. We argue that our calculated spectra may provide a better estimation of the scattering properties of whole blood than the compiled spectra from literature for wavelengths <600 nm.

## Background

### Composition of human blood and its optical properties

Normal human blood consists of red blood cells (RBCs or erythrocytes, ±4,500 × 10^3^/μL blood), white blood cells (leukocytes, ±8 × 10^3^/μL blood), platelets (thrombocytes, ±300 × 10^3^/μL blood) and blood plasma (containing water, electrolytes, plasma proteins, carbohydrates, lipids and various extracellular vesicles [18, 19]). The haematocrit (hct) is defined as the volume percentage of red blood cells in blood and on average amounts to 40 % for adult women and 45 % for adult men. Red blood cells are composed mainly of haemoglobin, with a concentration of ±350 g/L in a cell volume of ±90 fL. In healthy human adults, the average haemoglobin concentration in blood accounts for 140 g/L in women and 155 g/L in men [19].

Accounting for an absorption contribution of two to three orders of magnitude higher than the other blood components, red blood cells are by far the most dominant absorbing element in the blood in the wavelength range of 250–1,100 nm [20]. Practically, all light absorption by the red blood cells is due to haemoglobin, which exhibits specific absorption features for its various derivatives: bound to oxygen (oxyhaemoglobin, HbO_2_), unbound to oxygen (deoxyhaemoglobin, Hb), bound to carbon monoxide (carboxyhaemoglobin), oxidized (methaemoglobin), fetal and more [4]. From these haemoglobin derivatives, oxyhaemoglobin and deoxyhaemoglobin are the most abundant types in healthy human adult blood. The oxygen saturation of blood is defined as the ratio of the HbO_2_ concentration to the total haemoglobin concentration, oxygen saturation (SO_2_) = [HbO_2_] / ([HbO_2_] + [Hb]), and amounts to ∼97.5 % in arterial blood and ∼75 % in venous blood [19]. Of all blood particles, red blood cells also predominate the scattering of blood with two to three orders of magnitude, arising from the difference in refractive index between red blood cells and the surrounding blood plasma [20].

Without the presence of red blood cells, plasma absorption in the 250–1,100-nm region is dominated by various proteins, nutritive compounds and/or pharmaceuticals, while plasma scattering is dominated by proteins and platelets [20]. Under pathological conditions, the absorption contribution of certain plasma proteins can become significant even in the presence of red blood cells, e.g. the absorption of bilirubin around 460 nm for jaundiced patients [21].

In the wavelength range beyond 1,100 nm, blood absorption is dominated by the absorption of water [7, 9]. Only when water is removed from the blood, several absorption features due to the presence of haemoglobin, albumin and globulin can be identified as small absorption peaks between 1,690 and 2,400 nm [22].

### Factors influencing the optical properties of blood

Since red blood cells are the main contributor to the optical properties of blood, their volume percentage (i.e. haematocrit), haemoglobin concentration and oxygen saturation directly influence the absorption and scattering properties of blood. Whereas the absorption coefficient *μ*_a_ is proportional to the haematocrit, the scattering coefficient *μ*_s_ saturates for hct > 10 %, i.e. *μ*_s_, is underestimated for high hct values with respect to a linear relationship between the two parameters [10]. Meinke et al. [10], in our opinion correctly, ascribed this saturation effect to a decrease of the mean distance between red blood cells, because it violates the assumption of independent single scattering. This group also reported non-linear deviations of *g* for hct > 10 %. See part II section of this paper for further discussion.

The scattering of blood is primarily caused by the complex refractive index mismatch between red blood cells and plasma. Although most measurements on the optical properties of blood are performed on blood samples where plasma has been replaced by saline/phosphate buffer, Meinke et al. [10, 20] measured that this affects the complex refractive index mismatch considerably, resulting in an overestimation of the scattering coefficient of 5.5–9.4 % with respect to red blood cells in plasma.

The principle of causality dictates that the real and imaginary parts of the complex refractive index are connected as expressed by the Kramers–Kronig relations. The imaginary part is proportional to the absorption coefficient, which in turn depends on the SO_2_. Thus, the real part of the complex refractive index is also SO_2_ dependent and so are the scattering properties [9, 17]. This influence is most prominent in the visible wavelength region where differences in *μ*_a_ due to changes in SO_2_ are high, leading to deviations up to 15 % in *μ*_s_ and 12 % in *g* between fully oxygenated and fully deoxygenated blood [9].

Various sources have reported that the shear rate due to blood flow [7, 13–16] and aggregate formation (e.g. rouleaux formation) [13, 23, 24] significantly influence the optical properties of blood due to non-Newtonian flow characteristics. Enejder et al. [13] measured a decrease in the absorption and reduced scattering of bovine blood of ∼3 % when increasing the average shear rate from 0 to 1,600 s^−1^, as well as a decrease in reduced scattering of 4 % when randomly oriented red blood cells form aggregates.

Other reported factors of influence on the optical properties are osmolarity [7], temperature [25, 26], inter-person variability [9] and pathologic disorders such as sickle cell anemia [27]. A special case is that for adults versus fetuses, whose blood is composed of different types of haemoglobin (adult versus fetal haemoglobin) that exhibit slight variations in their absorption features [4].

### Measurement methods in literature

Most measurements on whole or diluted blood with intact red blood cells have been performed using single or double integrating sphere geometries. The resulting wavelength-dependent transmission and/or reflectance from a thin sample slab is analysed by inverse Monte Carlo models [6–10] or T-matrix computations [13] to obtain estimates for *μ*_a_, *μ*_s_ and *g*. As is acknowledged by various sources [6–8], the assumed scattering phase function of blood in the inverse Monte Carlo analysis highly influences the inferred optical properties—especially *μ*_s_ and *g*. Although other measurement methods have been reported for optical property measurements on whole blood [28, 29], we did not encounter any studies that exploit these methods experimentally or the quantitative assessment of spectra of *μ*_a_, *μ*_s_ and *g*.

In addition to whole blood measurements, non-scattering haemolysed blood has been investigated in conventional transmission measurement geometries to assess the *μ*_a_ of haemoglobin only [4, 5].

The refractive index of oxygenated haemoglobin solutions was determined by Friebel et al. [30] from measurements of the Fresnel reflection with an integrating sphere spectrometer. Complementing these measurements, Meinke et al. [10] measured the refractive index of plasma at four wavelengths using an Abbe refractometer, which yielded a Sellmeier equation for the visible wavelength range.

## Part I: the absorption coefficient of whole blood

### Methods

From the available optical property spectra in literature, we compiled the averaged spectra of *μ*_a_ for whole blood with a haematocrit of 45 %. Criteria for including optical property data were (1) publication of both absolute and spectrally resolved data on the optical properties and (2) the use of human blood from healthy adults for sample preparation. In case tabulated data were unavailable, the program *GetData Graph Digitizer* (v2.25.0.32) was used to obtain the digitized optical property spectra from the published graphs. The same criteria were applied for the inclusion and tabulation of literature spectra for *μ*_s_ and *g*, which will be considered in part II of this article.

#### Compiled literature spectrum of *μ*_a_

All spectra were resampled to a 1-nm increment wavelength axis. Depending on the description of sample concentration in hct or total haemoglobin (tHb), the *μ*_a_ spectra were rescaled to a hct of 45 % or an equivalent tHb concentration of 150 g/L. Linear rescaling of *μ*_a_ spectra with respect to hct = *X*%, i.e. *μ*_a,hct_ = 45 % *=* (45/*X*) and *μ*_a,hct_ = *X*%, yields incorrect results if the absorption by the medium (water or plasma) cannot be neglected. This leads to an overestimation of the *μ*_a_ spectra at wavelengths where water absorption is substantial (*λ* > 1,100 nm). We therefore perform a correction for the water absorption on the linearly rescaled *μ*_a_ spectra:

1 $$ {\mu}_{a, hct=45\%}\kern0.5em =\kern0.5em \frac{45}{X}\left({\mu}_{\mathsf{a},\mathsf{hct}= X\%}-{\mu}_{{\mathsf{a},\mathsf{H}}_2\mathsf{O}}{\left[{f}_{\mathsf{blood}}\right]}_{\mathsf{hct}= X\%}\right)+{\mu}_{{\mathsf{a},\mathsf{H}}_2\mathsf{O}}{\left[{f}_{\mathsf{blood}}\right]}_{\mathsf{hct}=45\%} $$

Here, *μ*_a,hct = 45 %_ is the rescaled *μ*_a_ spectrum to 45 % hct, *μ*_a,hct *= X*%_ is the literature *μ*_a_ spectrum at *X*% hct and *μ*_a,H2O_ is the absorption coefficient of pure water, for which we used the spectrum from Hale et al. [31]. The water volume fraction [*f*_blood_] in blood with hct = *X*% is obtained using:

2 $$ {\left[{f}_{\mathsf{blood}}\right]}_{\mathsf{hct}= X\%}=\left(1-\frac{X}{100}\right){f}_{\mathsf{plasma}}+\frac{X}{100}{f}_{\mathsf{RBC}} $$

where *f*_plasma_ and *f*_RBC_ are the water volume fractions in blood plasma and red blood cells, respectively. In our analysis, we used *f*_plasma_ = 0.90 and *f*_RBC_ = 0.66, which correspond to normal physiological water concentrations in plasma and red blood cells [7]. Equations 1 and 2 show that the correct scaling between haematocrits at a given wavelength depends on the absorption coefficient of water at that wavelength.

For the absorption spectra that were measured on non-scattering homogeneous haemoglobin solutions, also the absorption flattening effect should be taken into account when rescaling the *μ*_a_ to that of whole blood. Citing Friebel et al. [8], the absorption flattening effect can be described as: ‘when light passes through a suspension of absorbing particles, such as blood, photons that do not encounter red blood cells pass unattenuated by absorption. As a consequence, the transmitted light intensity is higher than it would be if all the haemoglobin were uniformly dispersed in the solution’, Duysens [32] quantitatively described the reduction of the absorption coefficient obtained from a suspension of particles, with respect to that of a solution in which in the same amounts of absorbing molecules are homogeneously distributed. Following the method of Duysens, adapting only the terminology, we arrive at:

3 $$ {\mu}_{\mathsf{a},\mathsf{blood}}=\left(\frac{1-{e}^{\left({\mu}_{\mathsf{a},\mathsf{Hb}}\cdot {d}_{\mathsf{RBC}}\right)}}{\mu_{\mathsf{a},\mathsf{Hb}}\cdot {d}_{\mathsf{RBC}}}\right){\mu}_{\mathsf{a},\mathsf{Hb}} $$

Where *μ*_a,blood_ and *μ*_a,Hb_ are the absorption coefficient of a blood sample and haemoglobin solution, respectively, and *μ*_a,RBC_ is the absorption coefficient of the hemoglobin solution inside the red blood cell. The last two absorption coefficients are related through the haematocrit, *μ*_a,Hb_ = *Hct*∙*μ*_a,RBC_. The length *d*_RBC_ is a typical dimension of a red blood cell. In this derivation, it was assumed that the RBCs can be represented by cubes with volume equal to an RBC (*d*_RBC_ = ^3^√90 μm). Following the same approach, Finlay and Foster [33] derived a more complex version of Eq. 3, valid for equivolumetric spherical particles. Since the difference between both forms is neglicable for the present parameters, we adhere to the much simpler form of Eq. 3 throughout this manuscript.

The compiled spectra of *μ*_a_ were obtained by averaging the rescaled spectra, with the exclusion of one outlier spectrum, as specified in the ‘Results’ section. The *μ*_a_ spectra for oxygenized (nine averages) and deoxygenized blood (three averages) were compiled separately.

### Results

#### Optical property spectra of human blood in literature

The available literature on optical property measurements within our inclusion criteria is summarized in Table 1, with the relevant information (based on the factors of influence that have been listed in the ‘Factors influencing the optical properties of blood’ section) that was available on measurement method and sample preparation. All spectra from samples with intact red blood cells were obtained using integrating sphere measurements in combination with inverse Monte Carlo simulations. Phase functions that were applied in the analysis of these literature spectra included the Henyey–Greenstein [6], the Gegenbauer–Kernel [7] and the Reynolds–McCormick phase function [8–10]; details can be found in the respective references. The Gegenbauer–Kernel and the Reynolds–McCormick phase function cited in these publications are the same [34]. Compiled spectra of the absorption coefficient of Hb and HbO_2_ solutions are available from Zijlstra [4] and Prahl [5].

**Table 1:** Literature on the optical properties of blood in the visible and near-infrared

Reference	Wavelength range (nm)	Method	Sample	Optical properties
Zijlstra et al. [4]	450–800	Transmission spectrophotometer	Hb solution from haemolysed RBCs (human); SO_2_ = 0, 100 %; *T* = 20–24 °C	*μ*_a_
Prahl [5]	250–1,000	Compiled data from Gratzer and Kollias	Hb solution from haemolysed RBCs; SO_2_ = 0, 100 %	*μ*_a_
Yaroslavsky et al. [6]	700–1,200	Double IS with inverse MC (*P*_HG_)	Fresh heparinized whole blood (human); hct = 45–46 %, SO_2_ > 98 %; no flow, *γ* = 0 s^−1^	*μ*_a_, *μ*_s_, *g*
Roggan et al. [7]	400–2,500	Double IS with inverse MC (*P*_GK_)	Fresh RBCs (human) in phosphate buffer; hct = 5 %; SO_2_ = 0, 100 %; in flow, *γ* = 500 s^−1^; *T* = 20 °C	*μ*_a_, *μ*_s_, *g*
Friebel et al. [8]	250–1,100	IS with inverse MC (*P*_RC_)	Fresh RBCs (human) in phosphate buffer; hct = 0.84, 42.1 %; SO_2_ > 99 %; in flow, *γ* = 600 s^−1^; *T* = 20 °C	*μ*_a_, *μ*_s_, *g*
Friebel et al. [9]	250–2,000	IS with inverse MC (*P*_RC_)	Fresh RBCs (human) in phosphate buffer; hct = 33.2 %; SO_2_ = 0, 100 %; in flow, *γ* = 600 s^−1^; *T* = 20 °C	*μ*_a_, *μ*_s_, *g*
Meinke et al. [10]	250–1,100	IS with inverse MC (*P*_RC_)	Fresh RBCs (human) in phosphate buffer and saline solution/plasma various samples between hct = 0.84 and 42.1 % (shown in Figs. 1, 3 and 4: hct = 8.6, 41.2 %); SO_2_ > 98 %; in flow, *γ* = 600 s^−1^; *T* = 20 °C	*μ*_a_, *μ*_s_, *g*

*P*_GK_ and the *P*_RC_ are identical phase functions [34].

*IS* integrating sphere, *MC* Monte Carlo, *P*_*HG*_ Henyey–Greenstein phase function, *P*_*GK*_ Gegenbauer–Kernel phase function, *P*_*RC*_ Reynolds–McCormick phase function, *RBCs* red blood cells, *hct* haematocrit, *SO*_*2*_ oxygen saturation, *γ* shear rate, *T* temperature, *μ*_a_ absorption coefficient, *μ*_s_ scattering coefficient, *g* anisotropy factor

^a^The *μ*_a_ spectra of Roggan are excluded from the compiled spectra, as discussed in ‘Compiled literature spectrum of *μ*_a_’ section

#### Compiled literature spectrum of *μ*_a_

**Fig. 1:**
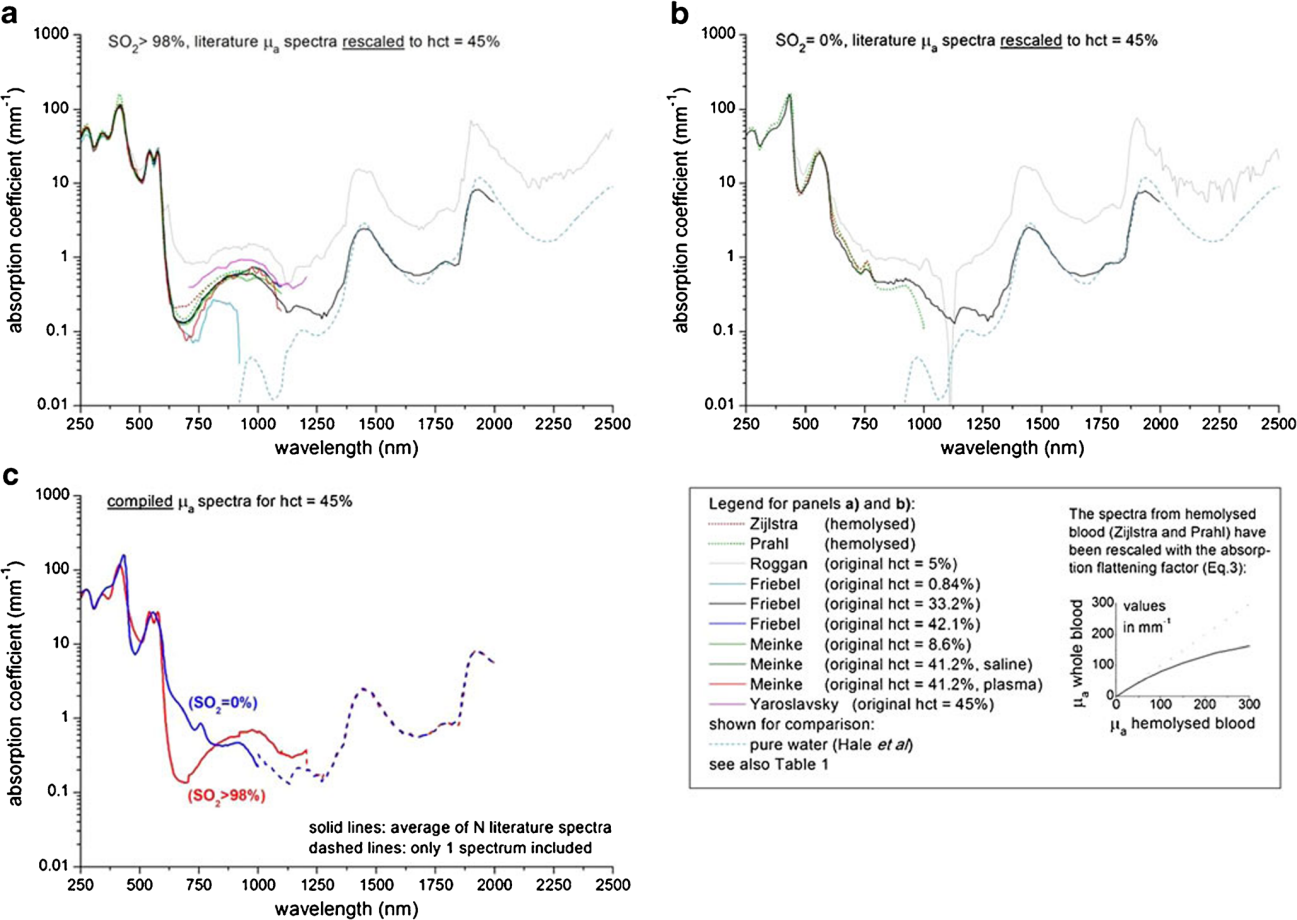
Blood absorption coefficient spectra from literature (see Table 1) within our inclusion criteria, rescaled to hct = 45 %: **a***μ*_a_ for oxygen saturation (SO_2_) > 98 %, **b***μ*_a_ for SO_2_ = 0 %, **c** compiled *μ*_a_ spectra for whole blood with a haematocrit of 45 %. The jumps around 1,000 and 1,200 nm in the compiled spectra are artifacts of our compilation method (caused by the transition of the average of multiple spectra to only one spectrum), which can be ignored or smoothed when using these spectra in practice. The spectra from haemolysed blood (Zijlstra and Prahl) in **a** and **b**) have been corrected for absorption flattening, see legend

Figure 1a, b displays the rescaled *μ*_a_ spectra to hct = 45 % for oxygenized blood (SO_2_ > 98 %) and deoxygenized blood (SO_2_ = 0 %), respectively. For both oxygenized and deoxygenized blood, the rescaled *μ*_a_ spectra of Roggan et al. [7] consistently overestimate the other *μ*_a_ spectra for nearly all wavelengths up to one order of magnitude. Roggan et al. obtained this overestimation with respect to pure haemoglobin and water solutions also for the original sample haematocrit of 5 %, and ascribed the difference to an increased probability of absorption due to elongated photon paths, resulting from internal photon reflections inside the red blood cells. When rescaling these values to hct = 45 %, the overestimation is magnified to unrealistically high values for *μ*_a_, in spite of the applied correction for the water absorption. We therefore excluded the *μ*_a_ spectra of Roggan et al. from the compiled spectra.

The spectra of haemolysed blood from Zijlstra [4] and Prahl [5] in Fig. 1a, b have been rescaled with the absorption flattening factor from Eq. 3. Unscaled, the *μ*_a_ spectrum of haemolised blood overestimates the absorption of both oxygenized and deoxygenized blood with approximately 10–20 % at the Soret band around 420 nm [5]. This difference has also been measured by Friebel et al. [8] when they compared their *μ*_a_ spectra from samples containing intact red blood cells to those containing haemolysed blood at exactly the same concentrations of haemoglobin. After correcting for the absorption flattening, the spectra are in good agreement with the absorption spectra from (whole) blood measurements.

The compiled *μ*_a_ spectrum of oxygenized blood is composed of the average of *N* = 9 spectra (Fig. 1c). Due to the difficulty to fully deoxygenize blood (high oxygen affinity of haemoglobin), fewer literature spectra are available for deoxygenized blood—resulting in a compiled *μ*_a_ spectrum of the average of *N* = 3 spectra for deoxygenized blood (Fig. 1c). Note that the data from Friebel et al. [9] are the only data contributing to the compiled spectrum beyond 1,200 nm for oxygenized blood and beyond 1,000 nm for deoxygenized blood (indicated by the dashed lines in Fig. 1c). The sudden jumps in the compiled spectra at 1,200 and 1,000 nm are caused by this transition of the average of multiple spectra to only one spectrum that differs slightly in amplitude (∼0.1 mm^−1^) from the other spectra. We consider these jumps as artifacts of our compilation method, which can be ignored or smoothed when using these spectra in practice.

## Part II: the scattering properties of whole blood

The determination of the scattering properties of whole blood is extremely challenging because assumptions on the applied scattering phase function are of high influence and the scaling of diluted blood measurements to physiological haematocrit values is not straightforward (‘Background’ section). In our previous work, we therefore proposed to use a ‘forward’ approach to estimate the light scattering properties from accurate measurements of the absorption coefficient of haemoglobin solutions, followed by Kramers–Kronig (KK) analysis and application of light scattering theory [17]. We expand on this theoretical approach here to include dependent scattering effects.

In the first step, the complex refractive index is determined from the absorption coefficient of the contents of one red blood cell. This is used as input to scattering theory in the second step, accounting for inter-particle correlations due to high-volume fractions. This way, the theoretical scattering property spectra of blood can be calculated for any haematocrit at any wavelength. We use this theory to obtain calculated spectra for *μ*_s_ and *g* for whole blood with a haematocrit of 45 %.

For practical convenience, we proceed to average the scaling factors for *μ*_s_ over wavelength, which leads to a simple expression depending on haematocrit only. This novel scaling relation is then used to rescale literature spectra of *μ*_s_ to a haematocrit of 45 %, from which we compile an average spectrum.

Summarizing, in this part II of the article, we provide both calculated and compiled literature spectra for *μ*_s_ and *g*. From this, we calculate the reduced scattering coefficient *μ*_s_′ and effective attenuation coefficient *μ*_eff_ for whole blood.

### Theoretical estimation of *μ*_s_ and *g*

#### Kramers–Kronig analysis

Causality dictates a functional relationship between the real and imaginary parts of the complex refractive index. This relation is expressed by the Kramers–Kronig integral dispersion equations. The imaginary part *κ*(*ω*) of the complex refractive index *m*(*ω*) = *n*(*ω*) + *iκ*(*ω*) is related to the absorption coefficient *μ*_a_ through:

4 $$ \kappa \left(\omega \right)=\frac{c{\mu}_{\mathsf{a}}\left(\omega \right)}{2\omega } $$

where *c* is the speed of light and *ω* is the angular frequency of the light. We use a subtractive KK equation [17, 35], so that:

5 $$ n\left(\omega \right)=n\left({\omega}_0\right)+\frac{2}{\pi}\left({\omega}^2+{\omega}_0^2\right)P{\displaystyle {\int}_0^{\infty}\frac{\omega \prime \kappa \left(\omega \prime \right)}{\left({\omega}^2+\omega \prime \right)\left({\omega}_0^2-\omega \prime \right)}} d\omega \prime $$

where *n*(*ω*_0_) is the refractive index at some reference frequency *ω*_0_, providing scaling of the calculated spectra. *P* denotes the Cauchy principle value of the integral. Thus, knowledge of the absorption spectrum of the haemoglobin solution inside an RBC, in combination with a reference value for the refractive index, allows determination of the complex refractive index of the solution at any given frequency *ω* (or wavelength *λ* = *c*/*ω*).

#### Scattering properties of red blood cells

The scattering properties of a single red blood cell (cross section and anisotropy) are calculated from the angularly resolved scattered intensity *I*_S_(*θ*), per unit input intensity [36]. The scattering cross section [in square metre] is given by:

6 $$ {\sigma}_{\mathsf{S}}=\frac{2\pi }{k^2}{\displaystyle {\int}_0^{\pi }{I}_{\mathsf{S}}\left(\theta \right) \sin \theta d\theta} $$

where *k* is the wave number *k* = 2*π*/*λ*. By normalization of *I*_S_(*θ*) on its 4*π* solid-angle domain, the *phase* function *p*_P_(*θ*) is obtained, which is parameterized by the expectation value of the cosine of the scattering angle, the scattering anisotropy *g* [−]:

7 $$ g=\frac{2\pi }{\sigma_{\mathsf{S}}{k}^2}{\displaystyle {\int}_0^{\pi } \cos \theta \cdot {I}_{\mathsf{S}}\left(\theta \right) \sin \theta d\theta} $$

These scattering properties can be calculated if an appropriate theory is available to calculate *I*_S_(*θ*). A common approach yielding reasonable agreement with experiment [37] is to describe the RBC as a sphere with an equivalent volume (90 μm^3^, ‘Composition of human blood and its optical properties’ section) using Mie theory.

#### Scattering properties of whole blood

We model light scattering of a blood medium by the angular resolved scattered intensity of a collection of *N* randomly distributed, identical particles:

8 $$ {I}_{\mathsf{S}}\left(\theta \right)=\left\langle {\displaystyle {\sum}_{m=1}^N{\displaystyle {\sum}_{n\kern0.3em =1}^N{E}_{\mathsf{S},m}{E}_{{}_{\mathsf{S},n}}^{*}{e}^{iq\left({r}_m-{r}_n\right)}}}\right\rangle $$

where E* denotes the complex conjugate of *E*. The ensemble average runs over all possible arrangements of the particles in volume *V*_T_ (that contains all particle contributing to the signal). *E*_s_,_*n*_ denotes the scattered field amplitude of the *n*th particle, located at r_*n*._ The scattering vector q has magnitude |*q*| = 2ksin(*θ*/2).

The terms *m = n* in the double sum define the light distribution when no interference between the scattered fields from different particles occurs, e.g. in a dilute medium. This condition is called ‘independent scattering’, and the total scattering cross section is simply *N* times the scattering cross section of a single particle. The scattering coefficient (or density of the scattering cross section, [in meter]) follows from *μ*_s_ = *σ*_s,TOTAL_/*V*_T_ or:

9 $$ {\mu}_{\mathsf{s},\mathsf{independent}}=N\frac{\sigma_{\mathsf{S}}}{V_{\mathsf{T}}}=\frac{\mathsf{hct}}{V_{\mathsf{P}}}{\sigma}_{\mathsf{S}} $$

with hct the particle volume fraction and *V*_p_ the particle volume.

If the particles are closely spaced, or when correlations between the particle positions are present, the interference effects cannot be ignored. This condition, usually called *dependent scattering* in the biomedical optics literature, takes into account the *m ≠ n* terms as well. Their contribution depends on the ordering in the arrangement of the particles, characterized by the radial distribution function *G*(*r*) which describes the probability of finding two particles spaced a difference *r* apart. We may write [38, 39]:

10 $$ \left\{\begin{array}{l}{I}_{\mathsf{S},\mathsf{dependent}}\left(\theta, hct\right)={I}_{\mathsf{S},\mathsf{independent}}\left(\theta \right)\cdot S\left(\theta, \mathsf{hct}\right)\\ {}S\left(\theta, \mathsf{hct}\right)=1+4\pi \frac{\mathsf{hct}}{V_{\mathsf{P}}}{\displaystyle {\int}_0^{\infty}\left\{G(r)-1\right\}{r}^2\frac{ \sin qr}{ qr} dr}\end{array}\right. $$

where |*q*| = 2ksin(*θ*/2). The term *S*(*θ*,hct) is called the structure factor, which thus allows to describe the angular scattering pattern from an ensemble of particles in terms of the scattered intensity pattern of a single particle, by applying a hct-dependent angular weighting of the scattered light. Combining Eq. 10 with Eq. 6, the scattering cross section for dependent scattering is found as:

11 $$ \left\{\begin{array}{l}{\sigma}_{\mathsf{S},\mathsf{dependent}}=\gamma \left(\mathsf{hct}\right){\sigma}_{\mathsf{S},\mathsf{independent}}\\ {}\gamma \left(\mathsf{hct}\right)=2\pi {\displaystyle {\int}_0^{\pi }S\left(\theta, \mathsf{hct}\right){p}_{\mathsf{P}}\left(\theta \right) \sin \theta d\theta}\end{array}\right. $$

where *γ*(hct) is the haematocrit-dependent scaling factor between the scattering cross section for dependent scattering and independent scattering, and *p*_P_(*θ*) is the single-particle phase function. The scattering coefficient follows as:

12 $$ {\mu}_{\mathsf{s},\mathsf{dependent}}=\frac{N}{V_{\mathsf{T}}}{\sigma}_{\mathsf{S},\mathsf{dependent}}=\frac{\mathsf{hct}}{V_{\mathsf{P}}}{\sigma}_{\mathsf{S},\mathsf{dependent}}=\gamma \left(\mathsf{hct}\right){\mu}_{\mathsf{s},\mathsf{independent}} $$

Expressions for the phase function and scattering anisotropy for dependent scattering can also be derived using the same methods.

Thus, the scattering properties of the blood medium can be calculated, provided a description for the radial distribution function *G*(*r*) is available (such as the Percus–Yevick model for non-deformable spheres used in this work). Scaling of the scattering coefficient between haematocrit values takes the following form for a blood medium:

13 $$ {\mu}_{\mathsf{s},\mathsf{dependent},\mathsf{hct}2}=\frac{\gamma \left(\mathsf{hct}2\right)}{\gamma \left(\mathsf{hct}1\right)}\frac{\mathsf{hct}2}{\mathsf{hct}1}{\mu}_{\mathsf{s},\mathsf{dependent},\mathsf{hct}1} $$

#### Practical formula for haematocrit dependent scaling of *μ*_s_

From the preceding analysis, it is clear that γ(hct)—the factor ultimately for non-linear scaling of the scattering coefficient with hct—can be a complicated function of wavelength because both *S*(*θ*,hct) and *p*_P_(*θ*) are wavelength dependent. However, some practical expressions for γ(hct), depending on haematocrit only, have been presented in the literature.

The best known is Twersky’s formula, which starts with the structure factor of Eq. 10 and employs a ‘small particle’ assumption replacing *S*(*θ*,hct) with *S*(0,hct) and uses *p*_P_(*θ*) = (4*π*)^−1^ [40–42] so that the integral over the radial distribution function *G*(*r*) is evaluated at *q* = 0 (or *θ* = 0). Assuming scatterers of radius *r*_p_ that do not attract or repulse each other (‘gas model’), we have *G*(*r*) = 0 (*r* ≤ *r*_p_); *G*(*r*) = 1 (*r* > *r*_p_). This leads to the simple expression:

14 $$ {\gamma}_{\mathsf{TWERSKY}\hbox{--} \mathsf{gas}}\left(\mathsf{hct}\right)=1-\mathsf{hct} $$

Following the same procedure using the radial distribution function of a collection of non-deformable small spheres gives:

15 $$ {\gamma}_{\mathsf{TWERSKY}\hbox{--} \mathsf{spheres}}\left(\mathsf{hct}\right)=\frac{{\left(1-\mathsf{hct}\right)}^4}{{\left(1+2\mathsf{hct}\right)}^2} $$

Both relations have been tested and have been found to be only in moderate agreement with experimental results on blood [11]. Based on their experiments, Steinke et al. therefore provide the following empirical relation:

16 $$ {\gamma}_{\mathsf{STEINKE}}\left(\mathsf{hct}\right)=\left(1-\mathsf{hct}\right)\left(1.4-\mathsf{hct}\right) $$

We compute *γ*(hct) at each wavelength using Eqs. 10 and 11 without restrictions on particle size, using the Mie phase function and the Percus–Yevick radial distribution function for non-deformable spheres. Averaging *γ*(hct, *λ*) over all wavelengths (250–2,000 nm) and both oxygenated forms and using a Levenberg–Marquardt non-linear least squares curve fitting procedure of *γ*(hct) versus hct yields the following approximation:

17 $$ {\gamma}_{\mathsf{MIE}-\mathsf{PY}}\left(\mathsf{hct}\right)=\left(1-\mathsf{hct}\right)\left(0.98\pm 0.02-\mathsf{hct}\right)\approx {\left(1-\mathsf{hct}\right)}^2 $$

For completeness, we give the equation relating the scattering coefficient of a blood sample (assuming dependent scattering) of given haematocrit to the scattering cross section of a single RBC as:

18 $$ {\mu}_{\mathsf{S},\mathsf{blood}}={\left(1-\mathsf{hct}\right)}^2\frac{\mathsf{hct}}{V_{\mathsf{RBC}}}{\sigma}_{\mathsf{S},\mathsf{RBC}} $$

Equations 17 and 18 are thus one of the main practical results of our work.

### Methods

#### Calculated spectra of *μ*_s_ and *g*

To compute the complex refractive index of an RBC’s contents, we model the RBC as a sphere (90 μm^3^), containing a homogeneous solution of haemoglobin molecules. Hereto, we use the average of the oxygenized and deoxygenized *μ*_a_ spectra of haemolysed blood from Prahl and Zijlstra only (part I)—rescaled to the appropriate concentration (350 g/L per RBC; ‘Composition of human blood and its optical properties’ 2.1), but not corrected for absorption flattening. Using Eq. 4, the imaginary part of the complex refractive index is obtained. In the Kramers–Kronig analysis (Eq. 5), we use a reference measurement of the real part of the complex refractive index at 800 nm to scale the computed spectra. Details of this procedure can be found in our previous publication [17]. The obtained complex refractive index spectra of oxygenized and deoxygenized blood are then used as input for subsequent calculations.

To implement the theory of Eqs. 6–13, a consistent combination of scattering theory and structure factor is needed. Here, we use the Mie theory [36] to calculate the scattered intensity and scattering properties by approximating a red blood cell with an equivolumetric sphere (*r* = 2.78 μm). Mie calculations also require specification of the refractive index of the medium in which the scattering particles are suspended (i.e. plasma). The refractive index of plasma has been determined experimentally by Streekstra et al. [43] at 633 nm and by Meinke et al. [9] at 400, 500, 600 and 700 nm. Since no data is available on the entire required wavelength range (including the near-infrared), we approximate the refractive index of plasma by that of water [31] with an additional offset to achieve a value of 1.345 at 633 nm [43]. This agrees well with the values of Meinke et al. in the visible wavelength range.

We use the Percus–Yevick approximation [44], solved analytically by Wertheim [45], to calculate the structure factor of a suspension of non-deformable spheres. The exact descriptions of the Percus–Yevick radial distribution function can be found elsewhere, e.g. in Refs. [39, 45]. All calculations are performed using self-written routines in Labview. The Kramers–Kronig code is benchmarked against the routines available from Ref. [35]; the Mie code is benchmarked against the results from Prahl’s web-based Mie calculator [46].

#### Compiled literature spectra of *μ*_s_ and *g*

The available literature on optical property measurements of *μ*_s_ and *g* within our inclusion criteria (‘Methods’ section) is summarized in Table 1. All spectra were obtained using integrating sphere measurements in combination with inverse Monte Carlo simulations. Phase functions that were applied in the analysis of these literature spectra included the Henyey–Greenstein [6], the Gegenbauer–Kernel [7] and the Reynolds–McCormick phase function [8–10]; details can be found in the respective references. The Gegenbauer–Kernel and the Reynolds–McCormick phase functions cited in these publications are the same [34].

We rescaled the *μ*_s_ spectra from their original haematocrits (hct = *X*%) to a whole blood haematocrit of 45 % using Eqs. 13 and 17. From the rescaled spectra (*N* = 8), we compiled an average spectrum. The compiled spectrum of the anisotropy *g* was obtained from the average of the unscaled literature spectra of *g* (*N* = 9).

#### Scatter power analysis on *μ*_s_

The scattering coefficient of most biological tissues exhibits a power law dependency on wavelength in the wavelength regions where *μ*_a_ is low with respect to *μ*_s_. This power dependency can be described by:

19 $$ {\mu}_{\mathsf{s}}=\mathit{\mathsf{a}}\cdot {\left(\frac{\lambda }{\lambda_0}\right)}^{-b} $$

with scaling factor *a* (in millimetre), scatter power *b* (dimensionless), scattering coefficient *μ*_s_ (in millimetre), wavelength *λ* and reference wavelength *λ*_0_. To summarize the obtained *μ*_s_ spectra of blood, we determined the parameters *a* and *b* for both the calculated and compiled spectra. Hereto, we fitted Eq. 19 to the spectra beyond 700 nm with a least squares algorithm, using *λ*_0_ = 700 nm. Error estimations were obtained from the 95 % confidence intervals of the fits.

#### Reduced scattering *μ*_s_′ and effective attenuation *μ*_eff_

In general, studies that rely on the diffuse reflectance or transmittance of whole blood consider the reduced scattering coefficient *μ*_s_′ = *μ*_s_(1 − *g*) and the effective attenuation coefficient *μ*_eff_ = √[3*μ*_a_(*μ*_a_ + *μ*_s_′)], rather than the scattering coefficient *μ*_s_ and absorption coefficient *μ*_a_ only. Therefore, we present the compiled spectra of *μ*_s_′ and *μ*_eff_, using the compiled spectra from literature for *μ*_a_, *μ*_s_ and *g*. We also present theory-derived spectra of *μ*_s_′ and *μ*_eff_, using the calculated spectra for *μ*_a_, *μ*_s_ and *g*, with *μ*_a_ obtained as *μ*_a_ = *μ*_ext_ − *μ*_s_ with *μ*_ext_ the calculated extinction coefficient from Mie theory.

### Results

#### Calculated spectra of *μ*_s_ and *g*

**Fig. 2:**
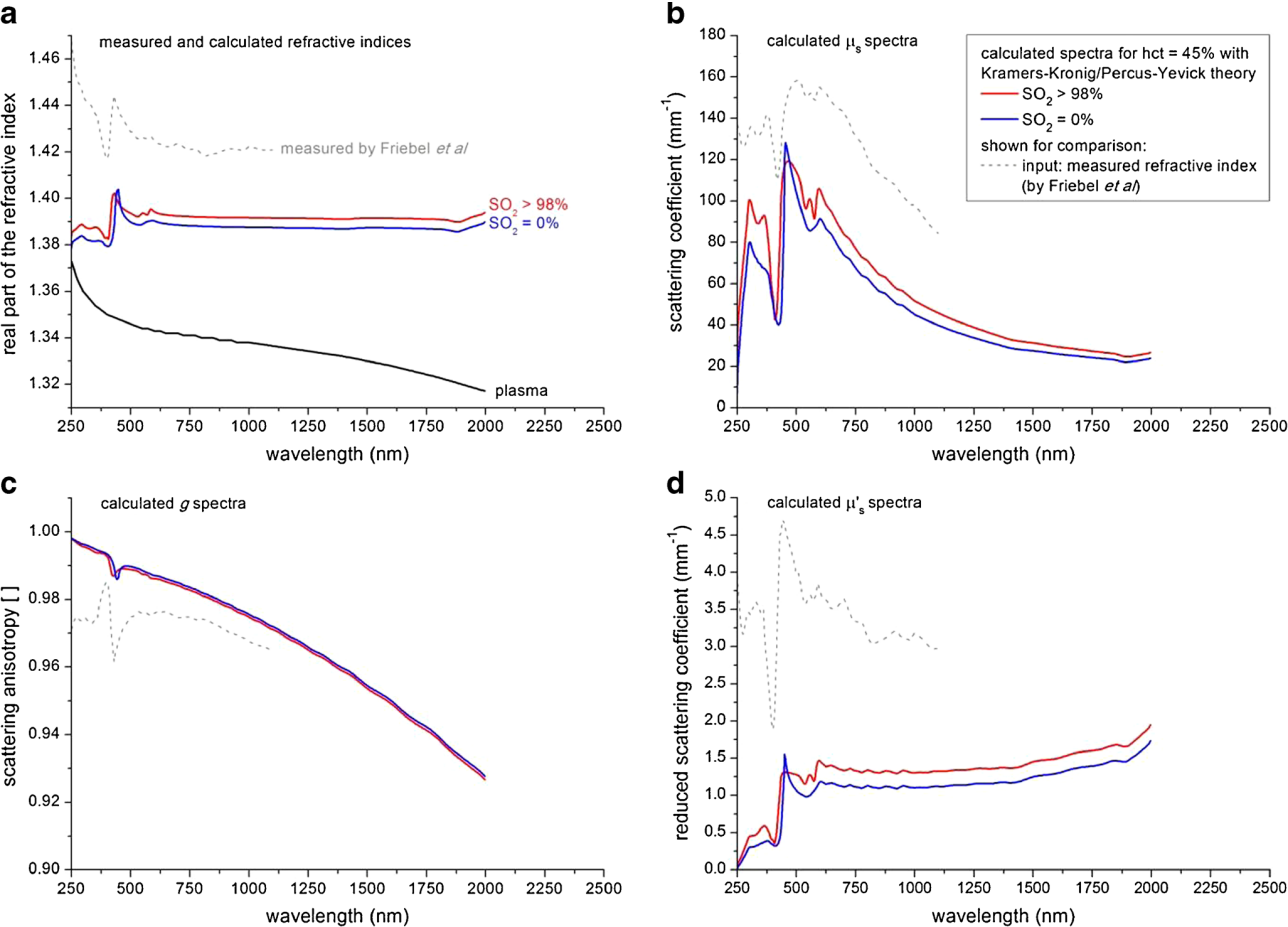
Theoretical analysis (Kramers–Kronig/Percus–Yevick) of the scattering properties of whole blood: **a** real part of the refractive index, **b** scattering coefficient, **c** scattering anisotropy (oxygenized and deoxygenized spectra overlap), **d** reduced scattering coefficient. For comparison, we also calculated the optical properties using the measured refractive index of aqueous haemoglobin (SO_2_ > 98 %) from Friebel et al. [30] (*grey lines*), instead of the calculated refractive index through the Kramers–Kronig analysis

Figure 2a shows the results of the Kramers–Kronig analysis to obtain the real part of the complex refractive index and for reference the experimental values obtained by Friebel et al. [30]. Also shown is the refractive index of plasma. Subpanels b and c of Fig. 2, respectively, show the calculated spectra of the scattering coefficient and anisotropy for both oxygenated and deoxygenated blood. Figure 2d shows the reduced scattering coefficient *μ*_s_′, obtained from the calculated spectra of *μ*_s_ and *g.* Results of the same calculations using the refractive index from Friebel et al. as input are also shown.

#### Compiled literature spectrum of *μ*_s_

**Fig. 3:**
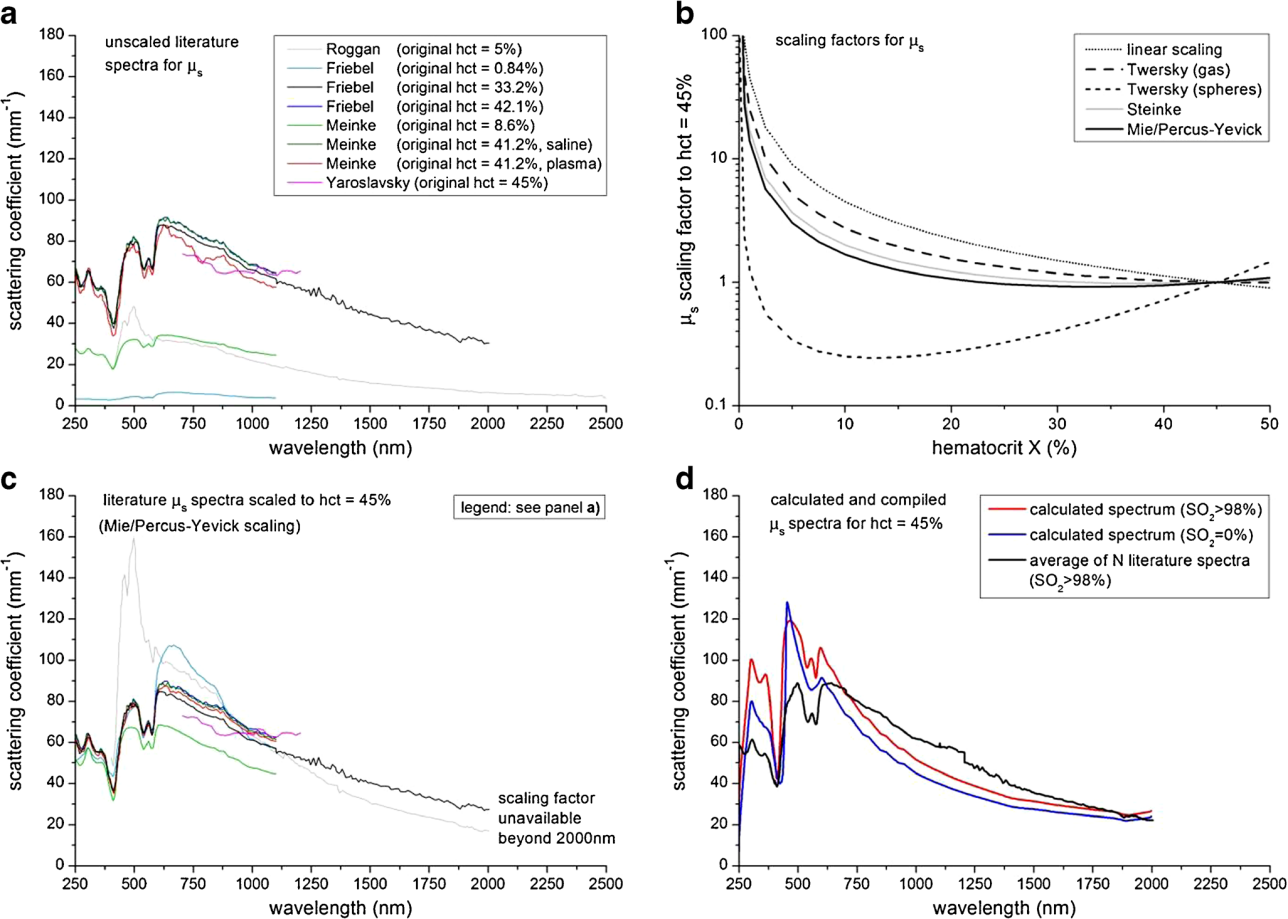
**a** Scattering coefficient spectra of blood from literature (see Table 1) within our inclusion criteria, displayed for their original hcts (hct = *X*%). **b** Scaling factors for rescaling *μ*_s_ at hct = *X*% to hct = 45 %: linear, according to Twersky for gas (Eqs. 13 and 14 combined) and spheres (Eqs. 13 and 15 combined) [40, 41], according to Steinke et al. (Eqs. 13 and 16 combined) [11] and our new scaling relation according to Mie/Percus–Yevick theory (Eqs. 13 and 17 combined). **c** Rescaling of the literature *μ*_s_ spectra (SO_2_ > 98 %) from their original hct (*X*%) to hct = 45 % using Eqs. 13 and 17. **d** Compiled *μ*_s_ spectrum for whole blood (SO_2_ > 98 %) with a haematocrit of 45 % (i.e. the average of the spectra in Fig. 3c). For comparison, also the calculated *μ*_s_ spectra from Fig. 2b are displayed. The jump around 1200 nm in the compiled *μ*_s_ spectrum is an artifact of our compilation method (caused by the transition of the average of multiple spectra to fewer spectra), which can be ignored or smoothed when using these spectra in practice

Figure 3a shows the unscaled literature spectra of *μ*_s_ at their original haematocrit values (all measured at SO_2_ > 98 %). To rescale these spectra to hct = 45 %, we apply our Mie/Percus–Yevick scaling relation (Eqs. 13 and 17 combined), which has been displayed in Fig. 3b. For comparison, also a linear scaling relation (hct/*X*) and the scaling relations of Twersky (Eqs. 13 and 14 combined and Eqs. 13 and 15 combined) [40, 41] and Steinke et al. (Eqs. 13 and 16 combined) [11] have been displayed. Figure 3c shows that the rescaled literature spectra using Eqs. 13 and 17 are much closer together in magnitude, compared to the unscaled spectra in Fig. 3a. The compiled average *μ*_s_ spectrum from the rescaled spectra (*N* = 8) agrees well in shape and magnitude with the calculated *μ*_s_ spectra for those wavelengths where scattering dominates absorption (beyond 700 nm, Fig. 3d). Comparable to the compiled spectrum of *μ*_a_, the jump in *μ*_s_ around 1,200 nm is an artifact of our compilation method, caused by the transition of the average of multiple spectra to fewer spectra.

#### Scatter power analysis on *μ*_s_

The scatter power analysis (Eq. 19) resulted in *a* values of 82.5 ± 0.2 mm^−1^ (calculated *μ*_s_, SO_2_ > 98 %), 72.2 ± 0.2 mm^−1^ (calculated *μ*_s_, SO_2_ = 0 %) and 91.8 ± 0.6 mm^−1^ (compiled *μ*_s_, SO_2_ > 98 %) using reference *λ*_0_ = 700 nm. The scatter power *b* values were 1.23 ± 0.005 (calculated *μ*_s_, SO_2_ > 98 %), 1.22 ± 0.006 (calculated *μ*_s_, SO_2_ > 0 %) and 1.19±0.012 (compiled *μ*_s_, SO_2_ > 98 %).

#### Compiled literature spectrum of *g*

**Fig. 4:**
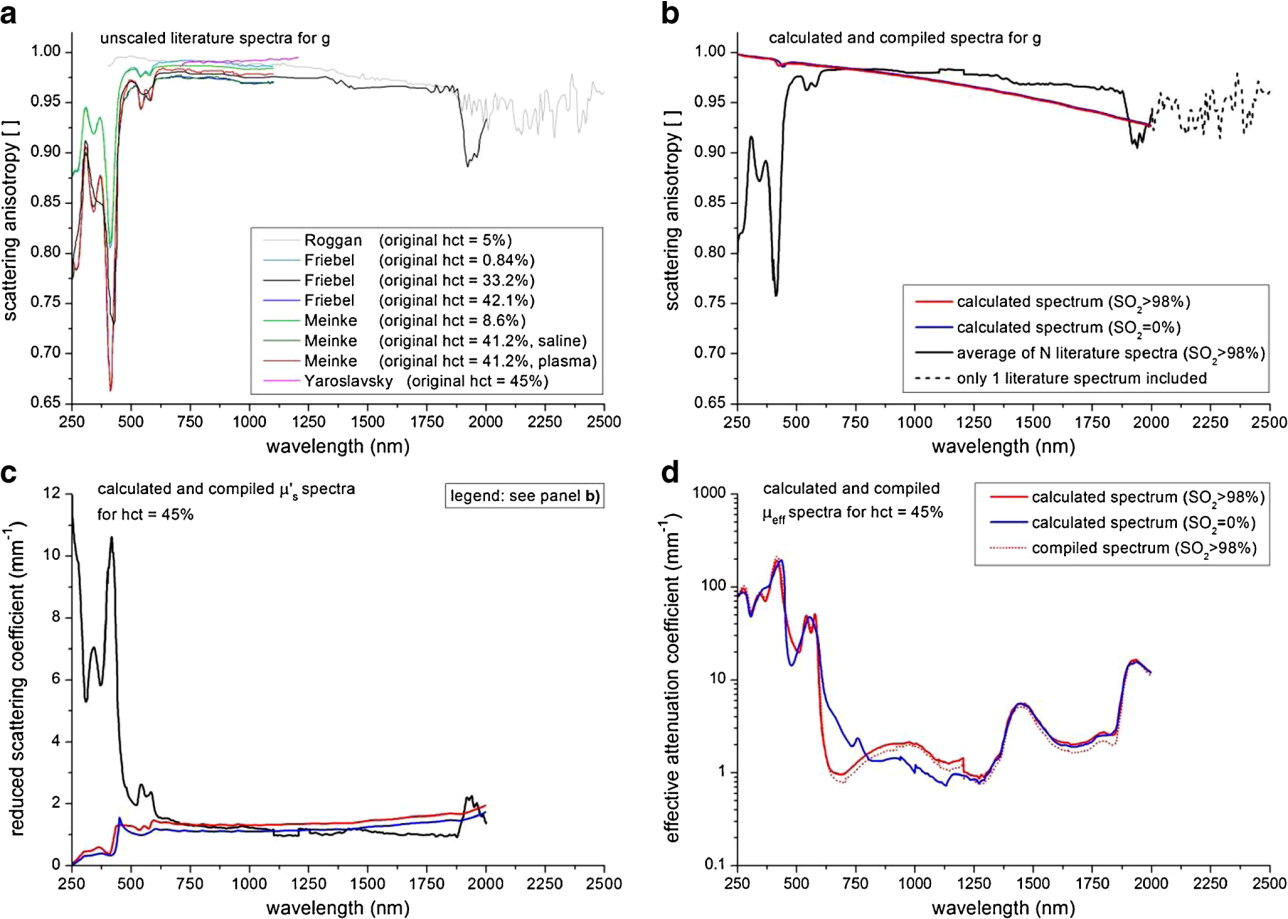
**a** Scattering anisotropy spectra of blood from literature (see Table 1) within our inclusion criteria, displayed for their original hcts. **b** Compiled spectrum for *g*; for comparison, also the *g* spectra from Fig. 2c are displayed. **c** Compiled *μ*_s_′ spectrum for whole blood with a haematocrit of 45 %; for comparison, also the *μ*_s_′ spectra from Fig. 2d are displayed. **d** Compiled and calculated *μ*_eff_ spectra spectrum for whole blood with a haematocrit of 45 %, for oxygenized (SO_2_ > 98 %) and deoxygenized blood (SO_2_ = 0 %)

Although a non-linear dependency of *g* on haematocrit has been reported (‘Background’ section), we do not perceive this effect in the literature spectra of *g* at the original haematocrit values (Fig. 4a). The compiled spectrum for *g* (Fig. 4b) is therefore obtained using all available, unscaled spectra (*N* = 9). Note that the data from Roggan et al. [7] are the only data contributing to the compiled spectrum beyond 2,000 nm (indicated by the dashed line in Fig. 4b). The large oscillations in *g* in this wavelength region are ascribed by Roggan et al. to ‘the small values of the measured quantities’, indicating a low signal-to-noise ratio for these wavelengths. Comparable to the compiled spectra of *μ*_a_ and *μ*_s_, the jump in *g* around 1,200 nm is an artifact of our compilation method, caused by the transition of the average of multiple spectra to fewer spectra. The compiled literature spectrum of *g* agrees well in magnitude with the calculated spectra of *g* for those wavelengths where scattering dominates absorption (beyond 700 nm).

#### Reduced scattering and effective attenuation

Figure 4c shows the calculated and compiled reduced scattering coefficient spectra *μ*_s_′, which were obtained using the calculated and compiled spectra of *μ*_s_ and *g*, respectively. Similar to the spectra of *μ*_s_ and *g*, the calculated and compiled *μ*_s_′ spectra agree well in magnitude for those wavelengths where scattering dominates absorption (beyond 700 nm).

Figure 4d shows the calculated and compiled effective attenuation coefficient spectra *μ*_eff_. For the calculated spectrum of *μ*_eff_, the absorption coefficient was calculated using Mie theory as the difference between the extinction coefficient and scattering coefficient (for both SO_2_ = 0 % and SO_2_ > 98 %). The compiled spectra were obtained using both the compiled spectrum for *μ*_a_ (part I) and the compiled spectrum for *μ*_s_′ (only for SO_2_ > 98 %). The absorption coefficient dominates *μ*_eff_. The excellent correspondence between the calculated and compiled spectra thus demonstrates that scattering theory is capable of including absorption flattening effects. The jumps in the 1,100–1,200 nm region and/or the oscillations beyond 2,000 nm in *μ*_s_′ and *μ*_eff_ are caused by the compilation artifacts in *μ*_a_, *μ*_s_ and *g* that have been explained above.

## Final remarks

### Tabulated data

In the Appendix of this article, we provide the tabulated data for the compiled spectra of *μ*_a_ (oxygenated and deoxygenated blood), *μ*_s_ and *g*. The table also includes the calculated spectra for *μ*_s_ and *g* (Kramers–Kronig/Percus–Yevick analysis for oxygenated and deoxygenated blood). All spectra are scaled to a haematocrit of 45 %. The data are presented with a resolution of 2 nm up to 600 nm and a resolution of 5 nm beyond 600 nm. From this, the calculated and compiled spectra for *μ*_s_′ and *μ*_eff_ can easily be calculated. The full table can also be downloaded at our website www.biomedicalphysics.org.

### Discussion

#### Compilation of optical property spectra from literature

In this article, we provided an overview of the available literature on the spectra of the optical properties (*μ*_a_, *μ*_s_ and *g*) of whole blood. Hereto, we included only data that present quantitative spectra of these properties and were measured on human blood or dilutions thereof. These restrictions limit the available data to the seven contributions as listed in Table 1, from which five contributions are obtained from (dilutions of) whole blood (*μ*_a_, *μ*_s_ and *g*), and two contributions are obtained from haemolysed blood (*μ*_a_ only). It should also be noted that experimental studies on the optical properties are scarce for wavelengths beyond 1,100 nm, compared to the visible and near-infrared wavelength range (*λ* < 1,100 nm). Hence, our compiled spectra beyond 1,100 nm are composed of only one (*μ*_a_ and *μ*_s_) or two (*g*) literature spectra, which makes them more susceptible to experimental or methodological errors than the compiled values for *λ* < 1,100 nm.

The compiled spectra for *μ*_s_ and *g* are largely dominated by the results from one research group (Roggan et al., Friebel et al. and Meinke et al. [7–10]), with three out of four literature spectra for *μ*_s_ and eight out of nine literature spectra for *g*. All spectra were obtained using integrating sphere setups, in combination with inverse Monte Carlo simulations (IS/iMC, to translate the measured diffuse reflectance and/or collimated and diffuse transmittance to values of *μ*_a_, *μ*_s_ and *g*). The results of the inverse procedure depend highly on the phase function that is used in the Monte Carlo simulations. For the research group of Roggan et al., Friebel et al. and Meinke et al., the preferred phase function is the Reynolds–McCormick (also called Gegenbauer–Kernel [34]) phase function. The authors argue that this phase function has better correspondence with their measurements than the often used Henyey−Greenstein phase function or the Mie phase function. This result can be understood considering Eq. 10, which shows that the ‘effective phase function’ of a blood medium is given by the single RBC phase function, multiplied with the concentration-dependent structure factor. An additional drawback of inverse Monte Carlo procedures is that all parameters are optimized independently, whereas, following from causality, a correlation exists between all optical properties (i.e. the Kramers−Kronig relations).

It would be beneficial to investigate the possibilities of other assessment techniques that avoid the methodological uncertainties (e.g. assumptions on phase function) that are associated with IS/iMC measurements. With optical coherence tomography (OCT), the non-diffusive component of the scattered light can be analysed, which facilitates quantification of the scattering properties, in addition to the absorption properties. With spectroscopic OCT [47, 48] and the closely related technique low-coherence spectroscopy (LCS), also the spectrally resolved optical properties can be quantified. LCS has been proven to give accurate estimations of *μ*_a_ and *μ*_s_ spectra in turbid media with relatively high attenuation (*μ*_a_ + *μ*_s_ up to 35 mm^−1^) both in vitro [49–51] and in vivo [52] in the visible wavelength range. Alternatively, methods that rely on the analysis of diffuse scattering from whole blood may be combined with other analysis models than the regular inverse Monte Carlo simulations.

#### Absorption flattening in whole blood: rescaling *μ*_a_

For the haemolysed blood spectra that contribute to the compiled spectrum of *μ*_a_ for whole blood, we take into account the absorption flattening effect. This effect involves the reduction of the absorption coefficient of a suspension of absorbing particles (i.e. blood containing RBCs), compared to a homogeneous solution containing the same number of absorbing molecules (i.e. haemolysed blood). The first theoretical assessment of absorption flattening originates from Duysens [32] for cubical-, spherical- and arbitrary-shaped particles. We use the cubical description (Eq. 3), since it only slightly deviates from Duysens’ more comprehensive spherical particle approximation (which was reintroduced by Finlay and Foster [33]). The analysis of Duysens assumes random placement of the absorbing particles, with no correlations between their positions (Poisson distribution; so that the spatial variance *σ*^2^ of the number of particles equals the mean number *μ* of particles). Applying Beer’s law to each of the particles (and unit transmission for the ‘holes’) leads to the result of Eq. 3 upon averaging over all possible particle arrangements. If all particles were stacked together, *σ*^2^ would be 0 (without changing *μ*) and the measured transmission would correspond to that of a homogeneous solution of the absorbing molecules—without absorption flattening. Thus, *σ*^2^ ultimately determines the flattening effect. In a whole blood medium, possible correlations between the particle positions lead to an increase in *σ*^2^. This causes a further reduction in the measured absorption coefficient [53]. Interestingly, the increased *σ*^2^ is determined by the volume integral of the radial distribution function *G*(*r*) [54], describing spatial arrangement that leads to dependent scattering effects (part II). This clearly emphasizes that the organization of a medium/tissue is reflected in all measurable optical properties. From a practical point of view, Duysens’ simple model of ‘cubic absorbers’ excellently scales data from haemoglobin solutions to the compiled absorption spectrum of blood.

The compartmentalization of haemoglobin in red blood cells causes the absorption flattening effect of blood absorption spectra compared to that of pure haemoglobin solutions. For techniques such as diffuse reflection spectroscopy, the same effect occurs on a larger scale because blood is not distributed homogeneously in tissue, but concentrated in vessels. Van Veen et al. [55] propose a correction factor introduced by Svaasand [56] that, interestingly, takes exactly the same form as Eq. 3 (but now with the vessel diameter as the length parameter instead of the diameter of the RBC), although it is derived in a completely different manner.

#### Dependent scattering in whole blood: rescaling *μ*_s_

All literature spectra of *μ*_s_ were rescaled to a haematocrit of 45 % in the compilation of the average spectrum, while taking into account the effect of dependent scattering. Dependent scattering occurs when particles (i.e. RBCs) are closely spaced, or correlations exist between their positions. In that case, the phase relation between the fields scattered from different particles cannot be neglected. Therefore, the scattered fields should be added, rather than the scattered intensities. We choose a numerical, forward approach to assess the effect of dependent scattering, in which we calculate the scattering properties of blood using Mie theory (independent scattering) and Mie theory in combination with the Percus–Yevick radial distribution function *G*(*r*) (dependent scattering). This choice of theoretical descriptions essentially models blood as a high-concentration suspension of non-deformable spheres. This approach does not do full justice to the structural and rheological complexity of RBCs and blood. Future work on scattering formalisms, such as discrete dipole approximations [57] or models for *G*(*r*), can thus be employed using the same methodology.

A main practical result of our work is the scaling factor *γ*(hct) that scales the scattering coefficients of independent scattering to dependent scattering. The most cited form in the literature is *γ*(hct) = 1 − hct (Eq. 14), proposed by Twersky [40]. However, in the derivation of this approximation, it is assumed that the scatterers are small and no correlations exist between their spatial positions—which is likely invalid for whole blood. Twersky’s scaling factor has been found in better agreement with experiments compared to linear haematocrit scaling (e.g. *γ*(hct) = 1), but other theoretical and empirical forms have been proposed, most importantly Eqs. 15 and 16. In this work, we propose *γ*(hct) = (1 − hct)^2^ as a practical approximation for the exactly calculated values from Mie/Percus–Yevick theory (Eq. 17). The agreement with the empirical form of Steinke et al. [11] is excellent.

#### Theoretical estimation of *μ*_s_ and *g*

In addition to the compiled spectra from literature, we also *calculated* the spectra of *μ*_s_ and *g* for whole blood, using only the absorption spectrum of blood as an input. The main advantage of this ‘forward approach’ to calculate these optical properties is that complicated measurements on whole blood are replaced by relatively straightforward absorption measurements on non-scattering haemoglobin solutions. However, both our calculations and whole blood measurements with IS/iMC require assumptions in the analysis method (as discussed for IS/iMC in ‘Compilation of optical property spectra from literature’ section). In our method, a choice for scattering theory and radial distribution function must be made.

Our calculated spectra of the scattering coefficient *μ*_s_ are in reasonable agreement with the compiled spectra from literature (Fig. 3d). The order of magnitude is the same over the whole wavelength range that is considered, and all spectral features occur at the same wavelengths. The largest deviations are found in the wavelength range where the absorption of blood is strong compared to its scattering (250–600 nm). The same discrepancies are found in the spectrum of the scattering anisotropy *g* (Fig. 4b). Interestingly, these deviations are less prominent in the compounded parameters *μ*_s_′ (reduced scattering coefficient, Fig. 4c) and *μ*_eff_ (effective scattering coefficient, Fig. 4d). Differences between the compiled and calculated spectra of *μ*_s_ and *g* may be caused either by false estimations of the phase function in the iMC analysis of the literature spectra and/or assumptions in our theoretical estimations.

In general, the input to Mie theory (or any other scattering formalism) is the complex refractive index *m*(*ω*) = *n*(*ω*) + *iκ*(*ω*) of the particle and of the suspending medium. In our calculations, its real part is obtained via Kramers–Kronig transformation of the imaginary part, which in turn is obtained from the absorption coefficient of haemoglobin (Eqs. 4 and 5). Meinke et al. [10] also calculated the scattering properties of blood using the Mie theory, using the experimentally determined values of the real part of the refractive index from haemoglobin solutions by Friebel et al. [30] (Fig. 2a). These measurements suggest that it can be expected that the refractive index of haemoglobin solutions will increase for wavelengths <300 nm, similar to the refractive index of plasma/water. This is not found in our calculations because haemoglobin absorption spectra (and thus of the imaginary part of the refractive index) are only available down to 250 nm. If these spectra would be available down to wavelengths overlapping with the water absorption in the UV, a similar increase in the refractive index is expected to be found. For this reason, we caution the use of our calculated spectra below 300 nm. However, the values for the refractive index of haemoglobin solutions from Friebel et al. [30] are on average 0.02 higher in magnitude than the values found through our Kramers−Kronig analysis (Fig. 2a), which Friebel et al. ascribed to sample preparation. Applying the experimentally determined refractive index of Friebel et al. in our analysis would therefore result in unrealistically high values for *μ*_s_ (Fig. 2b).

#### Choosing between the compiled and calculated spectra

Since the primary aim of this review is to provide the reader with a set of optical property spectra for whole blood that can be used in the practice of biomedical optics, the question remains which spectra the reader should choose from the provided results. For *μ*_a_, we present only compiled literature spectra of oxygenated blood and deoxygenated whole blood (Fig. 1c). Hence, our logical advice is to use these compiled spectra. For *μ*_s_, *g*, *μ*_s_′ and *μ*_eff_ however, we present both the compiled and the calculated Kramers–Kronig/Percus–Yevick spectra (Figs. 3d and 4b–d, respectively). The compiled spectra, as well as the calculated spectra rely on individual assumptions in their analysis. At present, we cannot assess which method provides the most reliable results. It is therefore difficult to draw any solid conclusions on the choice between the compiled and calculated spectra for *μ*_s_, *g*, *μ*_s_′ and *μ*_eff_.

In the wavelength below 600 nm, both the calculated and compiled experimental spectra of *all* optical properties show strong spectral fluctuations. This is expected, since the optical properties are strong functions of the complex refractive index. The real (*n)* and imaginary part (*κ*) of this quantity are interdependent on grounds of causality and as expressed by the Kramers–Kronig relations. The spectrum of *κ* can be easily obtained from the well-established absorption spectrum of haemoglobin solutions using Eq. 4. The spectrum of *n* is available through calculations (this work) and has been determined experimentally [30] as shown in Fig. 2a. Both methods show fluctuations in *n*(*λ*) around the large absorption peaks of haemoglobin with a modulation depth of 0.01–0.05 around their respective baseline values. To the best of our knowledge, no scattering theory applied to blood (cells) predicts the magnitude of the fluctuations in the compiled literature spectra of *μ*_s_ and particularly *g* using these input values. We hypothesize that this is largely due to the inverse Monte Carlo procedures as discussed in the ‘Compilation of optical property spectra from literature’ section. We therefore argue that our calculated spectra may provide a more consistent estimation of *μ*_s_, *g*, *μ*_s_′ and *μ*_eff_ for the wavelength range of 300–600 nm.

### Conclusion

In this article, we provided a critical review, compilation and calculation of the optical properties of whole blood (hct = 45 %). An important conclusion from our review study is that the optical properties of blood are influenced by a large variety of factors of both physical and methodological origin (‘Factors influencing the optical properties of blood’ section). One should always be aware of these factors when relying on literature spectra of *μ*_a_, *μ*_s_ and *g* or when performing one’s own optical property measurements on blood.

For two important factors of influence—the effects of absorption flattening and dependent scattering—we provided practical formulas for rescaling literature spectra that have been obtained from haemolysed and diluted blood, respectively. Our theoretically derived formula for the influence of dependent scattering on *μ*_s_ is in good agreement with the previously reported empirical relation by Steinke et al. [11].

The main results of this article are the compiled spectra for the *μ*_a_ of oxygenized and deoxygenized whole blood (Fig. 1c) and both the compiled and calculated spectra for *μ*_s_ (Fig. 3d), *g* (Fig. 4b), *μ*_s_′ (Fig. 4c) and *μ*_eff_ (Fig. 4d) of whole blood. We argue that our calculated spectra may provide a better estimation of *μ*_s_, *g*, *μ*_s_′ and *μ*_eff_ in the wavelength range of 300–600 nm. The compiled and/or calculated spectra of *μ*_a_, *μ*_s_ and *g* have been tabulated in the Appendix of this article. From that, the spectra for *μ*_s_′ and *μ*_eff_ can be easily calculated. With that, we hope that we have provided the reader with a set of optical property spectra for whole blood that can be used in the practice of biomedical optics.

## Appendix: Tabulated data for the optical property spectra of whole blood, hct = 45 %

Explanation of symbols:

*μ*_a_: Absorption coefficient
SO_2_: Oxygen saturation
*μ*_s_: Scattering coefficient
*g*: Scattering anisotropy

From these data, the reduced scattering coefficient (*μ*_s_′) and the effective attenuation coefficient (*μ*_eff_) can be calculated, using either the compiled, or the theoretical spectra (with SO_2_ > 98 % or SO_2_ = 0 %) as input:

$$ {\mu}_{\mathrm{s}}\prime ={\mu}_{{}_{\mathrm{s}}}\left(1\hbox{--} g\right) $$

$$ {\mu}_{\mathrm{eff}}=\surd \left[3{\mu}_{\mathrm{a}}\left({\mu}_{\mathrm{a}}+{\mu}_{\mathrm{s}}\prime \right)\right] $$

**Table:**

	Compiled averages from literature spectra				Kramers–Kronig/Percus–Yevick theory			
Wavelength	*μ*_a_, SO_2_ > 98 %	*μ*_a_, SO_2_ = 0 %	*μ*_s_, SO_2_ > 98 %	*g*, SO_2_ > 98 %	*μ*_s_, SO_2_ > 98 %	*μ*_s_, SO_2_ = 0 %	*g*, SO_2_ > 98 %	*g*, SO_2_ = 0 %
[nm]	[mm^−1^]	[mm^−1^]	[mm^−1^]	[−]	[mm^−1^]	[mm^−1^]	[−]	[−]
251	44.02	48.32	58.64	0.8088	22.47	7.00	0.9980	0.9981
252	42.29	48.89	58.61	0.8126	36.69	16.92	0.9978	0.9980
254	43.58	49.47	58.35	0.8147	41.34	21.01	0.9977	0.9979
256	44.89	50.26	58.00	0.8164	44.97	24.41	0.9976	0.9978
258	46.38	50.95	57.36	0.8187	48.07	27.57	0.9975	0.9978
260	47.89	51.57	56.99	0.8200	50.90	30.60	0.9974	0.9977
262	49.48	52.19	56.60	0.8202	53.52	33.55	0.9973	0.9976
264	51.00	52.81	56.04	0.8202	56.00	36.45	0.9972	0.9976
266	52.22	53.32	55.26	0.8204	58.41	39.31	0.9971	0.9975
268	53.29	53.83	54.94	0.8205	60.81	42.15	0.9970	0.9974
270	54.13	54.34	54.63	0.8210	63.40	45.01	0.9969	0.9973
272	54.59	54.63	54.35	0.8222	66.14	48.01	0.9967	0.9972
274	54.99	54.32	54.42	0.8235	68.80	51.06	0.9966	0.9971
276	54.97	53.92	54.63	0.8251	71.26	53.47	0.9965	0.9970
278	54.63	53.56	54.82	0.8281	73.30	55.28	0.9964	0.9970
280	54.14	53.19	54.99	0.8324	75.64	57.11	0.9963	0.9969
282	53.34	52.77	55.18	0.8374	77.96	59.03	0.9962	0.9968
284	52.30	52.03	55.38	0.8428	80.44	61.16	0.9961	0.9967
286	50.45	50.67	55.56	0.8497	82.95	63.32	0.9960	0.9967
288	48.22	48.72	56.00	0.8578	85.69	65.87	0.9960	0.9966
290	46.13	45.94	56.72	0.8648	87.56	68.80	0.9959	0.9965
292	43.58	43.08	57.56	0.8713	90.11	71.14	0.9958	0.9965
294	40.50	40.29	58.46	0.8798	93.61	73.39	0.9957	0.9964
296	37.82	37.45	59.36	0.8889	96.38	76.05	0.9957	0.9964
298	35.12	34.81	60.11	0.8976	98.85	78.16	0.9956	0.9963
300	32.52	33.15	60.64	0.9035	100.27	79.57	0.9956	0.9963
302	30.99	32.12	61.01	0.9084	100.50	79.87	0.9955	0.9962
304	30.01	31.25	61.36	0.9142	100.33	80.00	0.9955	0.9962
306	29.69	30.47	61.59	0.9159	99.79	79.89	0.9955	0.9962
308	29.75	30.28	61.28	0.9165	99.31	79.22	0.9954	0.9961
310	30.03	30.95	60.80	0.9159	98.78	78.45	0.9954	0.9961
312	30.73	32.10	60.24	0.9146	97.73	77.50	0.9953	0.9961
314	31.49	33.25	59.59	0.9129	96.70	76.71	0.9953	0.9960
316	32.45	34.43	58.90	0.9088	95.68	76.03	0.9952	0.9960
318	33.76	35.61	58.15	0.9029	94.81	75.40	0.9952	0.9959
320	35.15	36.86	57.50	0.8975	93.96	74.72	0.9951	0.9959
322	36.61	38.22	56.85	0.8938	93.08	74.02	0.9950	0.9958
324	38.03	39.50	56.31	0.8901	92.22	73.43	0.9950	0.9957
326	39.49	40.67	55.96	0.8861	91.40	72.96	0.9949	0.9957
328	40.94	41.70	55.59	0.8830	90.62	72.64	0.9948	0.9956
330	42.29	42.61	55.22	0.8797	89.98	72.41	0.9947	0.9955
332	43.46	43.51	54.73	0.8773	89.49	72.12	0.9947	0.9955
334	44.48	44.45	54.40	0.8758	89.26	71.74	0.9946	0.9954
336	45.42	45.94	54.19	0.8743	89.25	71.05	0.9945	0.9954
338	46.02	47.50	53.98	0.8735	89.20	70.45	0.9944	0.9953
340	46.46	48.90	53.80	0.8727	89.17	70.12	0.9943	0.9952
342	46.86	49.89	53.71	0.8720	89.22	70.02	0.9942	0.9951
344	47.23	50.81	53.63	0.8719	89.37	69.86	0.9942	0.9951
346	47.14	51.73	53.59	0.8724	89.94	69.68	0.9941	0.9950
348	46.73	52.68	53.54	0.8734	90.44	69.44	0.9940	0.9949
350	46.29	53.76	53.59	0.8750	90.96	69.11	0.9939	0.9949
352	45.80	54.73	53.84	0.8770	91.25	68.58	0.9939	0.9948
354	45.24	55.68	54.09	0.8793	91.48	68.10	0.9938	0.9947
356	44.37	56.62	54.37	0.8816	91.90	67.72	0.9938	0.9947
358	43.36	57.20	54.70	0.8841	92.56	67.64	0.9937	0.9946
360	42.38	57.74	54.59	0.8863	92.92	67.52	0.9937	0.9945
362	41.67	58.10	54.34	0.8881	93.05	67.39	0.9937	0.9945
364	41.22	58.43	54.08	0.8897	93.03	67.25	0.9937	0.9944
366	40.86	58.76	53.68	0.8913	92.66	67.07	0.9936	0.9944
368	40.72	59.10	53.23	0.8920	92.06	66.84	0.9936	0.9943
370	41.14	59.44	52.88	0.8922	90.92	66.57	0.9936	0.9943
372	42.09	59.74	52.49	0.8915	89.19	66.26	0.9936	0.9942
374	43.14	60.04	52.00	0.8902	87.52	65.93	0.9936	0.9941
376	44.26	60.34	51.33	0.8879	85.78	65.56	0.9936	0.9941
378	45.87	60.67	50.59	0.8851	83.85	65.15	0.9936	0.9941
380	47.86	61.28	49.79	0.8798	81.57	64.71	0.9935	0.9940
382	50.38	61.73	48.64	0.8741	79.10	64.17	0.9935	0.9940
384	54.31	62.83	47.42	0.8675	76.48	62.97	0.9935	0.9940
386	57.72	64.26	46.07	0.8603	73.63	61.59	0.9935	0.9939
388	62.58	65.75	44.93	0.8474	70.24	60.16	0.9934	0.9939
390	68.33	67.75	43.94	0.8379	66.26	58.39	0.9934	0.9938
392	73.62	71.01	43.03	0.8257	62.72	56.43	0.9932	0.9937
394	79.07	74.09	42.33	0.8127	59.85	54.90	0.9931	0.9937
396	84.00	76.95	41.77	0.7967	57.77	53.61	0.9929	0.9936
398	88.56	79.78	41.17	0.7863	56.09	52.39	0.9928	0.9935
400	93.07	82.54	40.12	0.7734	54.32	51.16	0.9926	0.9934
402	97.40	85.59	41.27	0.7866	52.31	49.55	0.9925	0.9933
404	101.82	89.21	40.25	0.7769	49.52	47.83	0.9924	0.9932
406	106.16	92.73	39.49	0.7726	45.77	46.47	0.9922	0.9930
408	110.73	96.09	38.90	0.7657	42.83	45.36	0.9916	0.9929
410	113.81	99.87	38.60	0.7604	42.44	44.39	0.9910	0.9927
412	116.09	104.21	38.95	0.7575	42.74	43.43	0.9904	0.9926
414	117.97	108.92	39.87	0.7585	43.62	42.46	0.9896	0.9924
416	118.04	114.03	41.09	0.7606	45.59	41.74	0.9890	0.9922
418	117.22	119.62	42.38	0.7657	47.68	41.18	0.9883	0.9919
420	115.39	125.10	43.91	0.7742	51.35	40.78	0.9876	0.9916
422	111.45	131.52	46.42	0.7896	56.08	40.43	0.9872	0.9914
424	106.30	139.37	49.90	0.8020	61.93	40.06	0.9870	0.9910
426	100.61	145.30	52.74	0.8141	68.03	40.40	0.9869	0.9906
428	94.94	151.01	55.61	0.8290	74.00	40.72	0.9870	0.9901
430	89.18	155.62	57.55	0.8390	79.94	40.89	0.9870	0.9896
432	82.69	159.31	60.51	0.8568	85.95	41.69	0.9871	0.9889
434	74.61	158.30	62.65	0.8710	95.79	43.48	0.9873	0.9882
436	66.95	156.93	64.74	0.8820	102.94	45.58	0.9877	0.9873
438	58.47	133.73	66.40	0.8928	105.63	50.05	0.9880	0.9865
440	51.07	118.33	68.83	0.9026	109.27	57.64	0.9882	0.9862
442	46.60	107.88	70.39	0.9220	111.29	63.54	0.9883	0.9860
444	42.24	88.84	72.82	0.9302	113.65	73.81	0.9885	0.9861
446	38.21	74.30	73.79	0.9343	114.47	81.66	0.9886	0.9862
448	34.26	54.74	75.05	0.9392	116.38	94.61	0.9887	0.9863
450	31.48	33.81	77.20	0.9447	116.55	118.29	0.9888	0.9869
452	29.42	25.94	78.01	0.9488	117.24	125.06	0.9888	0.9878
454	27.66	19.82	78.82	0.9520	118.02	128.16	0.9889	0.9884
456	26.00	17.04	79.42	0.9544	118.52	127.52	0.9889	0.9888
458	24.47	13.61	80.17	0.9567	118.65	126.75	0.9890	0.9890
460	22.82	11.45	80.31	0.9587	118.94	125.60	0.9890	0.9892
462	21.33	10.37	80.37	0.9611	119.16	124.50	0.9890	0.9894
464	20.10	9.64	80.85	0.9634	119.13	123.31	0.9890	0.9895
466	18.97	9.12	81.74	0.9651	119.26	122.08	0.9890	0.9896
468	17.93	8.65	82.77	0.9665	119.26	120.90	0.9890	0.9897
470	17.05	8.29	82.61	0.9677	119.15	119.73	0.9891	0.9897
472	16.30	7.99	83.21	0.9684	119.01	118.60	0.9891	0.9898
474	15.71	7.79	83.50	0.9690	118.85	117.42	0.9891	0.9898
476	15.16	7.60	83.81	0.9695	118.63	116.30	0.9891	0.9898
478	14.63	7.44	84.99	0.9700	118.38	115.20	0.9890	0.9898
480	14.15	7.31	86.13	0.9705	118.12	114.14	0.9890	0.9898
482	13.67	7.43	86.48	0.9714	117.80	113.05	0.9890	0.9899
484	13.30	7.56	86.75	0.9723	117.42	112.00	0.9890	0.9899
486	12.92	7.69	86.80	0.9731	117.03	110.99	0.9890	0.9899
488	12.54	7.86	86.81	0.9738	116.65	110.00	0.9890	0.9899
490	12.23	8.10	87.02	0.9743	116.26	108.96	0.9890	0.9898
492	11.94	8.35	87.52	0.9748	115.89	107.96	0.9889	0.9898
494	11.65	8.60	88.31	0.9752	115.52	106.99	0.9889	0.9898
496	11.37	8.87	88.75	0.9756	115.13	106.04	0.9889	0.9898
498	11.19	9.17	88.87	0.9759	114.73	105.10	0.9888	0.9898
500	11.05	9.50	88.63	0.9761	114.28	104.16	0.9888	0.9898
502	10.90	9.88	88.23	0.9762	113.83	103.25	0.9888	0.9897
504	10.74	10.25	87.75	0.9762	113.35	102.36	0.9888	0.9897
506	10.59	10.64	86.35	0.9762	112.88	101.49	0.9888	0.9897
508	10.52	10.99	85.75	0.9762	112.32	100.65	0.9887	0.9896
510	10.55	11.41	85.43	0.9763	111.71	99.82	0.9887	0.9896
512	10.74	11.91	85.03	0.9764	111.05	99.01	0.9887	0.9896
514	11.09	12.42	84.54	0.9765	110.29	98.21	0.9886	0.9895
516	11.59	12.92	83.74	0.9765	109.41	97.44	0.9886	0.9895
518	12.34	13.43	82.61	0.9763	108.32	96.68	0.9886	0.9894
520	13.26	13.98	81.42	0.9760	107.15	95.93	0.9886	0.9894
522	14.36	14.50	79.88	0.9756	105.81	95.16	0.9886	0.9894
524	15.62	15.22	78.06	0.9751	104.37	94.37	0.9885	0.9893
526	17.17	16.04	76.69	0.9744	102.87	93.59	0.9885	0.9893
528	19.20	16.96	75.28	0.9728	101.37	92.83	0.9884	0.9892
530	20.91	17.82	73.83	0.9707	99.91	92.08	0.9883	0.9892
532	22.48	18.66	72.78	0.9687	98.65	91.34	0.9883	0.9891
534	23.91	19.51	71.83	0.9668	97.67	90.60	0.9882	0.9891
536	25.17	20.37	71.10	0.9650	96.93	89.88	0.9881	0.9890
538	26.22	21.25	70.59	0.9637	96.44	89.18	0.9880	0.9890
540	27.13	22.13	70.14	0.9627	96.27	88.50	0.9879	0.9889
542	27.31	23.19	69.93	0.9625	96.43	87.87	0.9878	0.9888
544	26.79	24.01	69.89	0.9625	96.98	87.30	0.9877	0.9887
546	26.06	24.77	70.34	0.9627	97.79	86.81	0.9876	0.9887
548	25.15	25.45	70.86	0.9633	98.80	86.45	0.9875	0.9886
550	23.86	25.99	71.49	0.9642	99.80	86.20	0.9874	0.9885
552	22.54	26.38	72.27	0.9652	100.46	85.89	0.9874	0.9884
554	21.34	26.70	73.12	0.9664	100.86	85.66	0.9874	0.9884
556	20.29	26.80	73.70	0.9677	100.98	85.56	0.9874	0.9883
558	19.55	26.71	74.10	0.9690	100.71	85.54	0.9874	0.9883
560	19.12	26.56	74.13	0.9694	100.25	85.59	0.9873	0.9882
562	19.47	26.00	73.63	0.9696	99.50	85.77	0.9873	0.9881
564	20.14	25.38	72.90	0.9693	98.42	85.97	0.9873	0.9880
566	21.04	24.74	72.04	0.9687	97.07	86.17	0.9873	0.9880
568	22.56	24.06	70.98	0.9681	95.53	86.39	0.9872	0.9879
570	24.38	23.29	70.02	0.9677	93.95	86.59	0.9872	0.9879
572	25.62	22.38	69.20	0.9674	92.53	86.76	0.9871	0.9878
574	26.70	21.43	68.82	0.9670	91.56	86.91	0.9870	0.9878
576	27.20	20.49	68.84	0.9666	91.44	87.14	0.9869	0.9878
578	26.87	19.64	69.09	0.9663	92.37	87.45	0.9867	0.9877
580	25.75	18.87	70.07	0.9662	94.28	87.72	0.9865	0.9876
582	23.29	18.08	72.11	0.9669	96.77	87.97	0.9864	0.9876
584	20.06	17.27	74.68	0.9680	99.53	88.24	0.9863	0.9875
586	16.51	16.33	77.42	0.9692	101.95	88.55	0.9862	0.9874
588	12.68	15.30	79.88	0.9708	103.81	88.96	0.9862	0.9874
590	9.72	14.21	81.67	0.9724	105.02	89.50	0.9862	0.9873
592	7.32	13.12	83.13	0.9743	105.69	90.03	0.9862	0.9872
594	5.66	11.92	84.67	0.9759	105.95	90.54	0.9862	0.9872
596	4.48	10.60	85.58	0.9774	105.94	90.98	0.9862	0.9871
598	3.60	9.29	86.21	0.9784	105.74	91.35	0.9862	0.9871
600	2.62	7.53	86.88	0.9794	105.49	91.56	0.9861	0.9871
605	1.51	5.55	87.89	0.9809	104.02	90.96	0.9861	0.9870
610	0.88	4.07	88.09	0.9815	102.49	90.12	0.9860	0.9869
615	0.63	3.27	88.09	0.9820	101.04	89.04	0.9860	0.9869
620	0.46	2.82	88.28	0.9823	99.64	87.85	0.9859	0.9868
625	0.35	2.48	88.50	0.9824	98.30	86.66	0.9859	0.9867
630	0.28	2.27	88.55	0.9826	97.50	86.01	0.9857	0.9866
635	0.24	2.10	88.63	0.9827	96.75	85.38	0.9856	0.9864
640	0.20	1.98	88.84	0.9827	96.01	84.75	0.9854	0.9863
645	0.17	1.89	88.55	0.9826	95.30	84.15	0.9853	0.9862
650	0.16	1.80	88.01	0.9825	94.61	83.56	0.9852	0.9861
655	0.16	1.71	87.72	0.9825	93.44	82.50	0.9851	0.9859
660	0.15	1.64	87.61	0.9826	92.29	81.45	0.9850	0.9858
665	0.14	1.58	87.51	0.9828	91.16	80.44	0.9849	0.9857
670	0.14	1.51	87.25	0.9832	90.06	79.45	0.9848	0.9856
675	0.14	1.43	86.82	0.9830	88.98	78.47	0.9847	0.9855
680	0.14	1.35	86.61	0.9831	87.91	77.51	0.9846	0.9854
685	0.14	1.26	86.57	0.9834	86.87	76.57	0.9845	0.9853
690	0.13	1.17	86.35	0.9835	85.84	75.63	0.9843	0.9852
695	0.13	1.10	86.18	0.9835	84.83	74.71	0.9842	0.9850
700	0.14	1.00	85.70	0.9836	83.86	73.82	0.9841	0.9849
705	0.14	0.93	83.70	0.9837	83.35	73.40	0.9840	0.9848
710	0.17	0.87	83.33	0.9841	82.87	73.00	0.9838	0.9847
715	0.17	0.80	82.99	0.9840	82.40	72.61	0.9837	0.9845
720	0.18	0.75	82.57	0.9839	81.94	72.22	0.9835	0.9843
725	0.19	0.72	82.18	0.9839	81.46	71.82	0.9833	0.9841
730	0.20	0.70	81.64	0.9839	80.57	70.99	0.9831	0.9840
735	0.21	0.70	81.09	0.9839	79.67	70.13	0.9831	0.9839
740	0.22	0.72	80.66	0.9839	78.78	69.29	0.9830	0.9838
745	0.23	0.76	80.40	0.9838	77.90	68.45	0.9829	0.9837
750	0.24	0.81	80.22	0.9837	77.04	67.62	0.9827	0.9836
755	0.26	0.85	79.85	0.9836	76.19	66.84	0.9826	0.9834
760	0.27	0.84	79.41	0.9835	75.36	66.10	0.9824	0.9832
765	0.29	0.80	78.93	0.9835	74.54	65.39	0.9823	0.9831
770	0.30	0.73	78.42	0.9833	73.73	64.69	0.9822	0.9830
775	0.31	0.66	78.00	0.9832	72.95	64.01	0.9821	0.9829
780	0.33	0.59	77.61	0.9832	72.59	63.73	0.9819	0.9827
785	0.34	0.54	77.19	0.9832	72.25	63.47	0.9818	0.9826
790	0.36	0.51	76.82	0.9832	71.92	63.20	0.9816	0.9824
795	0.37	0.48	76.77	0.9831	71.59	62.94	0.9814	0.9822
800	0.38	0.47	76.73	0.9833	71.27	62.68	0.9812	0.9820
805	0.39	0.46	76.47	0.9832	70.53	62.00	0.9810	0.9818
810	0.40	0.45	76.10	0.9833	69.80	61.33	0.9809	0.9817
815	0.42	0.44	75.70	0.9833	69.08	60.66	0.9808	0.9816
820	0.43	0.44	75.48	0.9834	68.36	60.00	0.9807	0.9815
825	0.45	0.43	75.27	0.9835	67.66	59.35	0.9806	0.9814
830	0.46	0.43	75.06	0.9835	66.96	58.72	0.9804	0.9812
835	0.47	0.43	74.83	0.9834	66.28	58.09	0.9803	0.9811
840	0.48	0.43	74.65	0.9834	65.61	57.48	0.9801	0.9809
845	0.48	0.43	74.51	0.9833	64.95	56.87	0.9799	0.9807
850	0.49	0.42	74.20	0.9832	64.33	56.30	0.9798	0.9806
855	0.51	0.43	73.58	0.9831	64.06	56.08	0.9796	0.9804
860	0.51	0.43	72.87	0.9831	63.82	55.88	0.9794	0.9803
865	0.52	0.43	72.43	0.9831	63.58	55.69	0.9793	0.9801
870	0.53	0.43	72.29	0.9830	63.35	55.50	0.9791	0.9799
875	0.55	0.43	72.12	0.9829	63.09	55.29	0.9789	0.9797
880	0.56	0.44	71.63	0.9828	62.50	54.76	0.9788	0.9796
885	0.56	0.45	70.79	0.9828	61.90	54.20	0.9786	0.9794
890	0.56	0.45	70.07	0.9826	61.30	53.66	0.9784	0.9792
895	0.56	0.46	69.41	0.9824	60.72	53.12	0.9782	0.9791
900	0.56	0.46	68.86	0.9824	60.14	52.59	0.9781	0.9789
905	0.57	0.47	68.35	0.9823	59.57	52.07	0.9780	0.9788
910	0.57	0.47	68.07	0.9820	59.00	51.56	0.9778	0.9787
915	0.57	0.47	67.82	0.9820	58.44	51.05	0.9777	0.9786
920	0.57	0.47	67.54	0.9819	57.89	50.55	0.9776	0.9784
925	0.65	0.46	67.24	0.9818	57.38	50.09	0.9774	0.9783
930	0.65	0.45	66.86	0.9815	57.18	49.93	0.9772	0.9780
935	0.65	0.44	66.42	0.9812	57.01	49.81	0.9770	0.9778
940	0.65	0.43	66.08	0.9811	56.84	49.66	0.9768	0.9776
945	0.65	0.41	65.67	0.9810	56.67	49.55	0.9766	0.9774
950	0.65	0.39	65.21	0.9808	56.47	49.40	0.9763	0.9772
955	0.65	0.38	64.78	0.9805	55.99	48.97	0.9762	0.9770
960	0.66	0.36	64.38	0.9801	55.48	48.51	0.9760	0.9769
965	0.68	0.34	63.95	0.9800	54.97	48.06	0.9759	0.9767
970	0.69	0.32	63.58	0.9799	54.48	47.62	0.9758	0.9766
975	0.69	0.31	63.20	0.9798	53.99	47.18	0.9756	0.9765
980	0.68	0.29	62.68	0.9798	53.51	46.74	0.9755	0.9763
985	0.67	0.26	62.27	0.9798	53.04	46.31	0.9754	0.9762
990	0.66	0.25	62.09	0.9798	52.58	45.89	0.9752	0.9760
995	0.65	0.23	62.00	0.9800	52.11	45.47	0.9750	0.9758
1,000	0.64	0.22	61.83	0.9801	51.66	45.04	0.9748	0.9756
1,005	0.66	0.31	61.69	0.9801	51.32	44.74	0.9746	0.9754
1,010	0.65	0.28	61.62	0.9801	51.01	44.46	0.9744	0.9752
1,015	0.63	0.28	61.57	0.9800	50.69	44.18	0.9742	0.9750
1,020	0.61	0.28	61.46	0.9800	50.38	43.90	0.9740	0.9748
1,025	0.60	0.27	61.18	0.9800	50.07	43.62	0.9738	0.9747
1,030	0.58	0.24	60.85	0.9800	49.76	43.35	0.9737	0.9745
1,035	0.56	0.24	60.49	0.9800	49.46	43.08	0.9736	0.9744
1,040	0.53	0.23	60.35	0.9800	49.16	42.81	0.9734	0.9743
1,045	0.50	0.22	60.31	0.9800	48.86	42.55	0.9733	0.9741
1,050	0.49	0.22	60.15	0.9800	48.56	42.28	0.9731	0.9739
1,055	0.48	0.22	59.87	0.9800	48.27	42.02	0.9729	0.9737
1,060	0.46	0.21	59.53	0.9799	47.97	41.77	0.9727	0.9735
1,065	0.45	0.20	59.26	0.9799	47.69	41.52	0.9724	0.9732
1,070	0.42	0.19	59.04	0.9799	47.41	41.27	0.9722	0.9730
1,075	0.40	0.19	58.73	0.9800	47.13	41.02	0.9720	0.9728
1,080	0.37	0.18	58.34	0.9802	46.85	40.78	0.9718	0.9726
1,085	0.35	0.17	58.08	0.9801	46.58	40.54	0.9716	0.9724
1,090	0.34	0.17	57.98	0.9802	46.30	40.30	0.9714	0.9722
1,095	0.34	0.16	57.89	0.9804	46.02	40.06	0.9712	0.9721
1,100	0.35	0.15	57.81	0.9797	45.74	39.82	0.9711	0.9719
1,105	0.33	0.15	57.58	0.9834	45.48	39.59	0.9709	0.9718
1,110	0.33	0.16	57.66	0.9832	45.21	39.36	0.9708	0.9716
1,115	0.33	0.15	57.67	0.9832	44.95	39.13	0.9706	0.9715
1,120	0.31	0.14	57.72	0.9832	44.69	38.90	0.9704	0.9713
1,125	0.32	0.14	57.53	0.9829	44.43	38.68	0.9703	0.9711
1,130	0.31	0.13	57.26	0.9828	44.18	38.45	0.9700	0.9709
1,135	0.30	0.15	57.78	0.9832	43.92	38.23	0.9698	0.9706
1,140	0.30	0.16	57.93	0.9835	43.67	38.01	0.9696	0.9704
1,145	0.29	0.18	57.72	0.9834	43.43	37.79	0.9694	0.9702
1,150	0.30	0.19	57.51	0.9832	43.18	37.58	0.9691	0.9699
1,155	0.31	0.21	57.33	0.9830	42.94	37.37	0.9689	0.9697
1,160	0.32	0.21	57.05	0.9829	42.69	37.16	0.9687	0.9695
1,165	0.32	0.21	56.82	0.9828	42.46	36.96	0.9685	0.9693
1,170	0.32	0.21	56.55	0.9827	42.22	36.76	0.9683	0.9691
1,175	0.32	0.21	56.21	0.9827	41.99	36.56	0.9681	0.9689
1,180	0.33	0.21	55.55	0.9827	41.76	36.36	0.9679	0.9688
1,185	0.33	0.21	55.41	0.9827	41.54	36.17	0.9677	0.9686
1,190	0.34	0.21	55.48	0.9827	41.31	35.97	0.9676	0.9684
1,195	0.35	0.20	55.37	0.9827	41.09	35.78	0.9674	0.9683
1,200	0.36	0.20	55.20	0.9826	40.88	35.59	0.9672	0.9681
1,205	0.37	0.20	55.16	0.9826	40.70	35.40	0.9671	0.9679
1,210	0.20	0.20	50.16	0.9784	40.48	35.22	0.9669	0.9677
1,215	0.20	0.20	49.98	0.9784	40.25	35.03	0.9667	0.9675
1,220	0.20	0.19	49.81	0.9784	40.04	34.85	0.9665	0.9673
1,225	0.19	0.19	49.43	0.9784	39.82	34.67	0.9663	0.9671
1,230	0.18	0.17	49.21	0.9784	39.61	34.49	0.9660	0.9668
1,235	0.18	0.17	49.25	0.9784	39.40	34.31	0.9658	0.9666
1,240	0.18	0.17	49.19	0.9783	39.19	34.13	0.9655	0.9664
1,245	0.18	0.17	47.97	0.9782	38.99	33.96	0.9653	0.9661
1,250	0.17	0.17	47.51	0.9781	38.79	33.78	0.9651	0.9659
1,255	0.17	0.17	48.18	0.9781	38.58	33.61	0.9648	0.9656
1,260	0.17	0.17	48.64	0.9783	38.38	33.44	0.9646	0.9654
1,265	0.17	0.15	47.36	0.9781	38.19	33.27	0.9643	0.9652
1,270	0.15	0.14	46.50	0.9775	37.99	33.10	0.9641	0.9650
1,275	0.16	0.15	47.09	0.9774	37.79	32.93	0.9639	0.9648
1,280	0.18	0.16	47.91	0.9778	37.59	32.76	0.9638	0.9646
1,285	0.17	0.16	47.13	0.9778	37.40	32.59	0.9636	0.9645
1,290	0.16	0.16	46.21	0.9778	37.21	32.43	0.9634	0.9643
1,295	0.18	0.18	45.77	0.9778	37.01	32.26	0.9633	0.9641
1,300	0.19	0.20	45.36	0.9779	36.82	32.09	0.9631	0.9640
1,305	0.20	0.21	44.79	0.9780	36.63	31.93	0.9629	0.9638
1,310	0.21	0.22	44.21	0.9776	36.44	31.76	0.9628	0.9636
1,315	0.22	0.23	43.95	0.9772	36.25	31.60	0.9626	0.9634
1,320	0.24	0.26	43.91	0.9769	36.06	31.43	0.9624	0.9632
1,325	0.26	0.29	43.64	0.9766	35.87	31.27	0.9622	0.9630
1,330	0.29	0.31	43.38	0.9768	35.68	31.11	0.9619	0.9627
1,335	0.32	0.34	43.85	0.9771	35.49	30.95	0.9617	0.9625
1,340	0.35	0.37	44.37	0.9775	35.31	30.80	0.9614	0.9622
1,345	0.37	0.37	44.02	0.9775	35.13	30.65	0.9611	0.9619
1,350	0.39	0.38	43.68	0.9771	34.95	30.49	0.9609	0.9617
1,355	0.42	0.41	43.00	0.9764	34.76	30.33	0.9606	0.9614
1,360	0.45	0.43	42.32	0.9754	34.58	30.17	0.9603	0.9611
1,365	0.50	0.51	41.09	0.9744	34.38	29.99	0.9601	0.9609
1,370	0.59	0.65	40.31	0.9736	34.18	29.81	0.9598	0.9607
1,375	0.72	0.78	40.80	0.9738	33.97	29.64	0.9596	0.9604
1,380	0.88	0.92	41.69	0.9741	33.77	29.48	0.9594	0.9602
1,385	1.07	1.05	41.21	0.9743	33.57	29.32	0.9592	0.9600
1,390	1.30	1.20	40.43	0.9741	33.37	29.16	0.9590	0.9599
1,395	1.46	1.35	39.59	0.9733	33.20	29.01	0.9588	0.9597
1,400	1.62	1.49	39.21	0.9725	33.04	28.86	0.9586	0.9595
1,405	1.78	1.71	39.09	0.9715	32.90	28.74	0.9585	0.9594
1,410	1.94	1.83	38.98	0.9707	32.78	28.63	0.9583	0.9592
1,415	2.04	1.94	39.09	0.9699	32.67	28.53	0.9581	0.9590
1,420	2.13	2.08	39.08	0.9695	32.56	28.43	0.9579	0.9588
1,425	2.24	2.27	38.20	0.9694	32.45	28.33	0.9577	0.9586
1,430	2.33	2.36	38.15	0.9697	32.35	28.26	0.9575	0.9584
1,435	2.38	2.43	38.11	0.9692	32.26	28.19	0.9573	0.9582
1,440	2.38	2.50	38.01	0.9686	32.18	28.12	0.9571	0.9579
1,445	2.40	2.51	37.53	0.9683	32.10	28.07	0.9568	0.9576
1,450	2.41	2.48	37.05	0.9685	32.02	28.01	0.9565	0.9574
1,455	2.40	2.44	37.24	0.9686	31.94	27.96	0.9563	0.9571
1,460	2.38	2.37	37.36	0.9685	31.86	27.91	0.9560	0.9568
1,465	2.40	2.35	37.11	0.9682	31.78	27.84	0.9557	0.9565
1,470	2.34	2.33	36.85	0.9684	31.72	27.78	0.9554	0.9562
1,475	2.27	2.29	36.77	0.9689	31.65	27.72	0.9551	0.9559
1,480	2.20	2.24	36.72	0.9693	31.59	27.67	0.9548	0.9556
1,485	2.06	2.17	36.32	0.9684	31.54	27.61	0.9545	0.9553
1,490	1.89	2.07	35.91	0.9681	31.48	27.57	0.9542	0.9550
1,495	1.78	1.95	35.70	0.9682	31.39	27.52	0.9539	0.9548
1,500	1.71	1.82	35.56	0.9683	31.31	27.46	0.9537	0.9545
1,505	1.63	1.73	35.19	0.9685	31.23	27.39	0.9534	0.9543
1,510	1.50	1.63	35.07	0.9687	31.15	27.32	0.9531	0.9540
1,515	1.39	1.54	34.99	0.9690	31.06	27.25	0.9529	0.9538
1,520	1.34	1.49	34.98	0.9693	30.97	27.18	0.9527	0.9536
1,525	1.27	1.42	34.88	0.9688	30.87	27.11	0.9525	0.9534
1,530	1.21	1.33	34.72	0.9683	30.78	27.04	0.9523	0.9532
1,535	1.15	1.23	34.54	0.9686	30.68	26.97	0.9521	0.9530
1,540	1.11	1.14	34.33	0.9688	30.59	26.90	0.9519	0.9528
1,545	1.07	1.09	34.11	0.9685	30.49	26.81	0.9517	0.9526
1,550	1.03	1.05	33.98	0.9683	30.40	26.73	0.9514	0.9524
1,555	0.97	1.01	33.90	0.9686	30.31	26.65	0.9513	0.9522
1,560	0.91	0.97	33.82	0.9683	30.22	26.57	0.9510	0.9520
1,565	0.85	0.92	33.73	0.9685	30.12	26.49	0.9508	0.9518
1,570	0.82	0.87	33.63	0.9694	30.02	26.42	0.9506	0.9515
1,575	0.79	0.82	33.53	0.9691	29.92	26.34	0.9504	0.9513
1,580	0.75	0.78	33.37	0.9688	29.82	26.25	0.9502	0.9511
1,585	0.73	0.75	32.93	0.9684	29.73	26.17	0.9499	0.9508
1,590	0.72	0.71	32.60	0.9683	29.63	26.09	0.9497	0.9506
1,595	0.70	0.69	32.55	0.9682	29.53	26.01	0.9495	0.9503
1,600	0.69	0.67	32.48	0.9681	29.43	25.93	0.9492	0.9501
1,605	0.67	0.65	32.44	0.9681	29.36	25.87	0.9489	0.9498
1,610	0.66	0.64	32.40	0.9682	29.28	25.81	0.9486	0.9495
1,615	0.64	0.62	32.32	0.9683	29.21	25.74	0.9483	0.9492
1,620	0.64	0.62	32.21	0.9684	29.14	25.68	0.9480	0.9489
1,625	0.63	0.61	31.76	0.9684	29.06	25.62	0.9477	0.9486
1,630	0.63	0.61	31.50	0.9685	28.99	25.56	0.9474	0.9483
1,635	0.62	0.61	31.38	0.9685	28.92	25.50	0.9471	0.9480
1,640	0.62	0.61	31.21	0.9685	28.85	25.45	0.9468	0.9477
1,645	0.62	0.60	31.08	0.9684	28.78	25.39	0.9465	0.9473
1,650	0.60	0.59	31.02	0.9682	28.71	25.33	0.9462	0.9470
1,655	0.58	0.57	31.10	0.9681	28.64	25.28	0.9459	0.9467
1,660	0.57	0.56	31.17	0.9679	28.57	25.22	0.9456	0.9465
1,665	0.57	0.56	30.97	0.9671	28.50	25.16	0.9453	0.9462
1,670	0.57	0.56	30.75	0.9663	28.42	25.10	0.9450	0.9459
1,675	0.57	0.56	30.48	0.9663	28.35	25.04	0.9447	0.9456
1,680	0.57	0.56	30.23	0.9663	28.28	24.98	0.9444	0.9454
1,685	0.57	0.57	30.02	0.9662	28.21	24.93	0.9442	0.9451
1,690	0.58	0.58	29.81	0.9660	28.14	24.87	0.9439	0.9449
1,695	0.59	0.58	29.66	0.9660	28.07	24.81	0.9437	0.9446
1,700	0.60	0.59	29.65	0.9660	28.00	24.75	0.9434	0.9444
1,705	0.60	0.59	29.75	0.9660	27.93	24.69	0.9432	0.9442
1,710	0.61	0.60	29.75	0.9660	27.86	24.64	0.9430	0.9440
1,715	0.61	0.61	29.40	0.9660	27.79	24.58	0.9428	0.9437
1,720	0.62	0.61	29.08	0.9659	27.72	24.52	0.9425	0.9435
1,725	0.62	0.62	28.89	0.9658	27.65	24.46	0.9423	0.9433
1,730	0.64	0.63	28.71	0.9657	27.58	24.41	0.9421	0.9431
1,735	0.65	0.64	28.55	0.9655	27.50	24.35	0.9419	0.9429
1,740	0.67	0.65	28.49	0.9653	27.43	24.29	0.9417	0.9427
1,745	0.69	0.66	28.56	0.9651	27.36	24.23	0.9415	0.9425
1,750	0.71	0.68	28.62	0.9650	27.29	24.17	0.9413	0.9423
1,755	0.74	0.72	28.43	0.9652	27.22	24.10	0.9411	0.9420
1,760	0.76	0.75	28.23	0.9649	27.15	24.04	0.9408	0.9418
1,765	0.78	0.79	28.00	0.9640	27.08	23.99	0.9406	0.9416
1,770	0.81	0.81	27.76	0.9633	27.01	23.93	0.9404	0.9413
1,775	0.83	0.82	27.59	0.9634	26.94	23.88	0.9401	0.9410
1,780	0.84	0.83	27.49	0.9635	26.87	23.82	0.9398	0.9407
1,785	0.86	0.84	27.55	0.9646	26.80	23.77	0.9395	0.9404
1,790	0.88	0.84	27.60	0.9663	26.74	23.71	0.9392	0.9401
1,795	0.88	0.84	27.37	0.9651	26.67	23.66	0.9389	0.9398
1,800	0.87	0.84	27.14	0.9639	26.61	23.60	0.9386	0.9395
1,805	0.86	0.83	26.89	0.9628	26.56	23.56	0.9383	0.9392
1,810	0.84	0.84	26.65	0.9629	26.51	23.51	0.9379	0.9388
1,815	0.83	0.84	26.55	0.9630	26.46	23.46	0.9376	0.9385
1,820	0.81	0.84	26.57	0.9639	26.40	23.41	0.9372	0.9381
1,825	0.79	0.85	26.65	0.9647	26.34	23.36	0.9369	0.9378
1,830	0.78	0.88	26.56	0.9643	26.27	23.29	0.9365	0.9374
1,835	0.78	0.90	26.33	0.9640	26.19	23.23	0.9362	0.9370
1,840	0.80	0.94	26.11	0.9630	26.11	23.15	0.9358	0.9367
1,845	0.80	0.97	25.97	0.9610	26.01	23.07	0.9355	0.9364
1,850	0.87	1.02	25.75	0.9622	25.89	22.97	0.9351	0.9360
1,855	1.10	1.21	25.52	0.9619	25.75	22.84	0.9348	0.9357
1,860	1.40	1.81	25.23	0.9610	25.59	22.68	0.9345	0.9355
1,865	1.78	2.19	24.79	0.9591	25.43	22.53	0.9342	0.9352
1,870	2.16	2.65	24.35	0.9597	25.26	22.41	0.9339	0.9349
1,875	3.03	3.24	23.96	0.9615	25.05	22.27	0.9337	0.9346
1,880	3.90	3.99	23.57	0.9597	24.89	22.13	0.9334	0.9344
1,885	4.60	5.05	23.77	0.9540	24.77	22.01	0.9332	0.9341
1,890	5.30	5.89	24.16	0.9479	24.68	21.96	0.9329	0.9339
1,895	6.16	6.56	24.03	0.9421	24.61	21.98	0.9326	0.9336
1,900	7.10	7.00	23.90	0.9361	24.58	22.02	0.9324	0.9333
1,905	7.40	7.33	23.96	0.9326	24.63	22.08	0.9321	0.9330
1,910	7.69	7.41	24.27	0.9244	24.69	22.18	0.9318	0.9327
1,915	8.01	7.41	24.48	0.9156	24.77	22.26	0.9315	0.9325
1,920	8.08	7.41	24.25	0.9096	24.86	22.33	0.9312	0.9322
1,925	8.13	7.57	24.15	0.9113	24.95	22.38	0.9309	0.9320
1,930	8.17	7.70	24.26	0.9122	25.04	22.44	0.9307	0.9317
1,935	8.22	7.84	24.03	0.9081	25.14	22.51	0.9304	0.9314
1,940	7.97	7.73	23.75	0.9052	25.27	22.62	0.9301	0.9312
1,945	7.79	7.57	23.54	0.9141	25.38	22.72	0.9298	0.9309
1,950	7.58	7.41	23.32	0.9197	25.49	22.82	0.9296	0.9306
1,955	7.34	7.25	23.06	0.9159	25.58	22.91	0.9293	0.9304
1,960	7.11	7.07	22.69	0.9110	25.68	23.00	0.9290	0.9301
1,965	6.87	6.85	22.63	0.9147	25.77	23.10	0.9287	0.9298
1,970	6.63	6.63	22.63	0.9206	25.86	23.19	0.9284	0.9295
1,975	6.40	6.41	22.32	0.9267	25.95	23.29	0.9282	0.9292
1,980	6.17	6.19	22.15	0.9282	26.05	23.38	0.9279	0.9289
1,985	6.00	6.04	22.24	0.9269	26.16	23.50	0.9275	0.9286
1,990	5.86	5.88	22.27	0.9291	26.30	23.64	0.9272	0.9282
1,995	5.72	5.73	22.26	0.9349	26.53	23.88	0.9268	0.9278
2,000				0.9370				
2,005				0.9291				
2,010				0.9249				
2,015				0.9366				
2,020				0.9390				
2,025				0.9404				
2,030				0.9448				
2,035				0.9560				
2,040				0.9568				
2,045				0.9539				
2,050				0.9438				
2,055				0.9479				
2,060				0.9526				
2,065				0.9545				
2,070				0.9545				
2,075				0.9532				
2,080				0.9506				
2,085				0.9466				
2,090				0.9449				
2,095				0.9481				
2,100				0.9512				
2,105				0.9535				
2,110				0.9522				
2,115				0.9418				
2,120				0.9311				
2,125				0.9255				
2,130				0.9219				
2,135				0.9236				
2,140				0.9238				
2,145				0.9204				
2,150				0.9191				
2,155				0.9233				
2,160				0.9399				
2,165				0.9430				
2,170				0.9413				
2,175				0.9348				
2,180				0.9255				
2,185				0.9245				
2,190				0.9257				
2,195				0.9341				
2,200				0.9381				
2,205				0.9424				
2,210				0.9434				
2,215				0.9291				
2,220				0.9198				
2,225				0.9311				
2,230				0.9544				
2,235				0.9442				
2,240				0.9335				
2,245				0.9427				
2,250				0.9611				
2,255				0.9627				
2,260				0.9609				
2,265				0.9521				
2,270				0.9420				
2,275				0.9418				
2,280				0.9431				
2,285				0.9275				
2,290				0.9145				
2,295				0.9311				
2,300				0.9422				
2,305				0.9499				
2,310				0.9525				
2,315				0.9550				
2,320				0.9569				
2,325				0.9576				
2,330				0.9583				
2,335				0.9551				
2,340				0.9443				
2,345				0.9426				
2,350				0.9433				
2,355				0.9616				
2,360				0.9746				
2,365				0.9751				
2,370				0.9664				
2,375				0.9648				
2,380				0.9639				
2,385				0.9426				
2,390				0.9218				
2,395				0.9218				
2,400				0.9258				
2,405				0.9363				
2,410				0.9433				
2,415				0.9391				
2,420				0.9286				
2,425				0.9334				
2,430				0.9545				
2,435				0.9532				
2,440				0.9524				
2,445				0.9589				
2,450				0.9702				
2,455				0.9692				
2,460				0.9645				
2,465				0.9624				
2,470				0.9607				
2,475				0.9612				
2,480				0.9616				
2,485				0.9596				
2,490				0.9581				
2,495				0.9598				
2,500				0.9613				
